# Geoarchaeological research on site formation process, paleoenvironment, and human behaviors in the early Holocene of the Gobi Desert, Mongolia

**DOI:** 10.1371/journal.pone.0330209

**Published:** 2025-09-02

**Authors:** Grzegorz Michalec, Rafał Sikora, Małgorzata Winiarska-Kabacińska, Davaakhuu Odsuren, Antoni Wójcik, Piotr Moska, Marcin Szmit, Dashzeveg Bazargur, Przemysław Bobrowski, Maciej Jórdeczka, Józef Szykulski, Patryk Muntowski, Andrzej Gałaś, Byambaa Gunchinsuren, Mirosław Masojć

**Affiliations:** 1 Institute of Archaeology, University of Wroclaw, Poland; 2 Polish Geological Institute – National Research Institute, Kraków, Poland; 3 Poznań Archaeological Museum, Poland; 4 Institute of Archaeology, Mongolian Academy of Science, Ulaanbaatar, Mongolia; 5 Mongolian National University of Education, Ulaanbaatar, Mongolia; 6 Institute of Physics, Division of Geochronology and Isotope Research of the Environmental, Silesian University of Technology, Gliwice, Poland; 7 Gdańsk Archaeological Museum, Poland; 8 Institute of Archaeology and Ethnology, Polish Academy of Sciences, Poznań Branch, Poland; 9 Scientia et Arte Foundation, Gdańsk, Poland; 10 Mineral and Energy Economy Research Institute, Polish Academy of Sciences, Kraków, Poland; University of Haifa, Zinman Institute of Archaeology, ISRAEL

## Abstract

This paper presents a rare example of the multi-proxy investigation results on the prehistoric settlement from vast areas of the Mongolian Gobi Desert, where, during favorable climatic conditions, postglacial hunter-gatherer groups occupied a seasonal lake district. The geoarchaeological research conducted at site FV92, located at the Luulityn Toirom Paleolake, provides insight into the problem of human relations with the changing environment of the Early Holocene, as well as the problem of the site formation process in the Gobi area. Sedimentological studies and luminescence dating of the Luulityn Toirom Lake sediments indicate the presence of the lake and favorable environmental conditions for human settlement in the Early Holocene in the period before 8130 ± 83 BP. Spatial analyses of the artifact distribution, as well as refitting studies of the discovered lithic assemblage, enabled the determination of the site’s formation process. Initially, the site was influenced by fluvial processes, but as the climate dried, it was subsequently affected by aeolian processes. The techno-typological analysis, refitting studies, and microscopic analyses carried out provide the first such detailed insight into the technological behavior and identification of the *chaîne opératoire* used by the Early Holocene hunter-gatherer communities of the Gobi area. The results confirmed that the lithic technology was mainly based on microblade technology. Microscopic analyses of traces created during tool use indicate butchery activity and the use of plant resources. The studies indicate a high degree of mobility of hunter-gatherer communities living by the lakes, as evidenced by the medium-range transport of raw material brought to the campsite from the surrounding mountainous Altai area.

## Introduction

The climatic and environmental changes that have occurred since the beginning of the Holocene have influenced the adaptation of the subsistence strategies of hunter-gatherer communities living in the area of the present-day Gobi Desert. Studies of lake sediments from Mongolian sites indicate highly humid environmental conditions in the early and middle Holocene, followed by a warming and drying of the environment thereafter [1–3]. The causes of these changes are attributed to two factors. In the Early Holocene, an extensive network of rivers and lakes was established as climatic conditions improved and permafrost thawed [1,4]. Evidence to date suggests that the climate in the Early Holocene was influenced by the East Asian Winter Monsoon, associated with the eastward and southward movement of cold air from the Siberian High [5]. Studies in the Gobi Desert of southern Mongolia and northern China have also demonstrated the influence of the East Asian Summer Monsoon System (EASM) in increasing environmental moisture since the climatic optimum, which occurred in the middle Holocene (~8,000–5,000 BP) [1,3,6–8].

The problem of human-environment relations during the Holocene of the Gobi area has attracted little research interest. The first comprehensive hypothesis on this research problem was presented by J. Maringer [9]. He postulated that the beginning of the Holocene favored human settlement (Mesolithic), which was associated with a warming climate and an extensive network of rivers and lakes created by the melting of glaciers. Subsequently, as the climate dried up, Neolithic communities inhabited areas adjacent to small oasis-type reservoirs. In recent years, issues of adaptation, subsistence strategies, and human-environment interactions in the Gobi region have been addressed by L. Janz [6,10]. An example of this work is the results of a study conducted at the Zaraa Uuul site in the Gobi area [11]. The results of this study showed that the area was mainly settled in different episodes from the late Pleistocene and middle Holocene. Of particular note are the geomorphological and palynological results for the period ~8500−6500 cal BP, which confirm the presence of a wet marsh environment with seasonal fluvial tributaries during the Middle Holocene in the area. This would suggest the influence of the EASM in improving environmental conditions conducive to human settlement.

Chronological data concerning human settlement and cultural changes in the Altai-Gobi region in the Early Holocene are still sketchy. The significant increase in data on newly discovered sites from the Gobi area occurred in the 1920s and 1930s with the activities of the Central Asian Expedition and the Sino-Swedish Expedition [9,10], as well as from the second half of the 20th century with the increase in activity of the Russian-American-Mongolian Archaeological Mission [12–15]. During this period, fieldwork methodologies differed from modern methods, and luminescence and radiocarbon dating methods were not commonly used. Most of the sites discovered so far are in a surface context, primarily due to the lack of sedimentation growth resulting from the aeolian processes of the Gobi Desert area. Only a few sites have been discovered in a stratified context. An example of such a site is the Chikhen Agui rock shelter [12]. Within this site, three archaeological horizons were identified, featuring numerous remains of hearths, concentrations of lithic artifacts, and bone beads. The original dating results indicate that the shelter was occupied from the late Pleistocene to the middle Holocene (11,545 ± 75–5,630 ± 220 BP). The most recent dating results of beads made from ostrich eggshells indicate seven episodes of cave settlement, with the oldest dating results pushing the earlier chronological findings back to 13,675–13,341 cal BP [15]. The technological features of the lithic assemblages have been linked to the ‘Mesolithic’.

The factors mentioned above also influenced the problem of determining the chronological phases of Holocene prehistoric communities of the Gobi area. Practically until the beginning of the 21st century, there was a simple classification into Mesolithic (hunter-gatherer communities functioning from the end of the Pleistocene, with no knowledge of pottery production methods, mainly using microblade cores in the production of stone tools) and Neolithic (semi-sedentary foragers groups in Holocene, which were technologically distinguished by the use of pottery, polished tools) [10]. The first more precise chronological frameworks were made by L. Janz [6,7,10,16,17]. Based on a techno-typological analysis of the lithic assemblages and using the C14 Accelerator Mass Spectrometry (AMS) dating method for ostrich eggshells and beads made from them, as well as luminescence dating for pottery fragments, she identified three main phases spanning from the late Pleistocene to the late Holocene. It should also be noted here that the dating of ostrich eggshell fragments indicates secondary use of their fossil forms by Holocene communities, making it challenging to date human activity using this method [17]. The oldest separate phase in the classification proposed by L. Janz – Oasis 1/Mesolithic - is dated to 13.5–8.0 cal kyr BP and concerns hunter-gatherer communities characterized by high mobility and living in wetlands [6,7,10]. Lithic assemblages for this phase are characterized primarily by the use of microblade technology and the production of geometric microliths, with occasional flake core production. Furthermore, around 9.6 cal kyr BP pottery production appears to have had poor firing and organic admixture.

The Oasis 2/Neolithic phase is then separated, with a chronological interval of 8.0 k-5.0 cal kyr BP, which describes communities with reduced residential mobility and a survival strategy focused on logistical foraging. Technological features distinguishing this phase include the continued presence of microblade technology, an increased proportion of flake core technologies, and the emergence of chipped and polished adze/axe-type tools, as well as grinding stones. There has been an increased proportion of ceramics on sites from this period, with better manufacturing technology for the type of textile-impressed pottery. The last separate phase (Oasis 3 – Eneolithic, Bronze Age) falls between 5.0–3.0 cal kyr BP. Regarding organization strategies, this period is characterized by Wetland-centric logistical foraging and the introduction of domesticated herd animals. The main technological features of the artifact assemblages for this phase include an increase in the use of flake cores and the presence of bifacially flaked arrowheads, blades, knives, milling stones, and copper slags. The main ceramic features are molded rim coarse redware, and geometrically incised wares.

In recent years, the Polish-Mongolian Expedition has been carrying out research in the Arts Bogdyn Nuruu area, which resulted in verification of the previous findings concerning the chronology of exploitation of raw material outcrops in the spacious massif abounding in rock raw material outcrops and prehistoric lithic workshops, referred to in the literature as the Flint Valley [18–23]. Archaeological survey revealed the existence of a network of paleolakes situated a few kilometers to the south of Arts Bogd, resulting in the discovery of numerous sites connected with the communities inhabiting the area in the Pleistocene as well as younger evidence of the presence of hunter-gatherer groups in the vicinity of the lakes in the early and middle Holocene [21,22,24].

In this article, we present the results of research on human activity and environmental conditions in the Early Holocene at site FV92, located in one of the five paleolakes present in the Flint Valley region of the Gobi Desert, specifically Lake Luulityn Toirom. The geoarchaeological results presented here provide a rare example of applying such a research approach in a discussion on adapting human survival strategies to environmental changes occurring in the Holocene in the Gobi area and the broader context of Northeast Asia. Analyses of the stone artifact assemblage uncovered at the site, based on techno-typological studies, refitting studies, and use-wear analyses, allowed for a more detailed examination of technological behavior and the identification of the operational chain used by mobile hunter-gatherer communities.

Considering the current state of research, the following main research questions were posed due to the above-mentioned factors. What environmental conditions prevailed in the area during human activity in the Early Holocene? What kind of site formation processes impacted the site; did only aeolian processes influence the state of preservation of the site? What was the function of the site? What was the technological behavior of the hunter-gatherer group inhabiting site FV92, and does it fit in with previously established chrono-technological phases of human activity in the Holocene of the Gobi Desert?

## Materials and methods

### Lake Luulityn Toirom – regional geographical and geological settings

**Fig 1:**
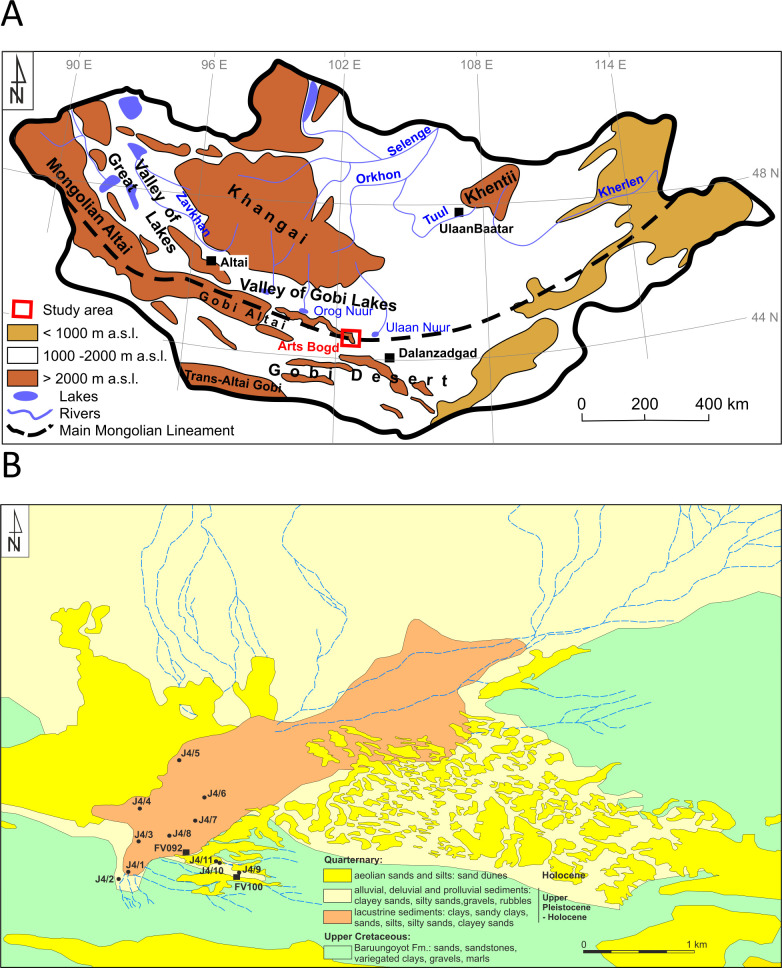
Geological structure of Mongolia and location of the sites (created by authors in ArcGIS PRO, and edited in GIMP 2.10.36. A) the location of the study area on the sketch map of Mongolia; B) the geological map of Lake Luulityn Toirom and adjacent areas.

**Fig 2:**
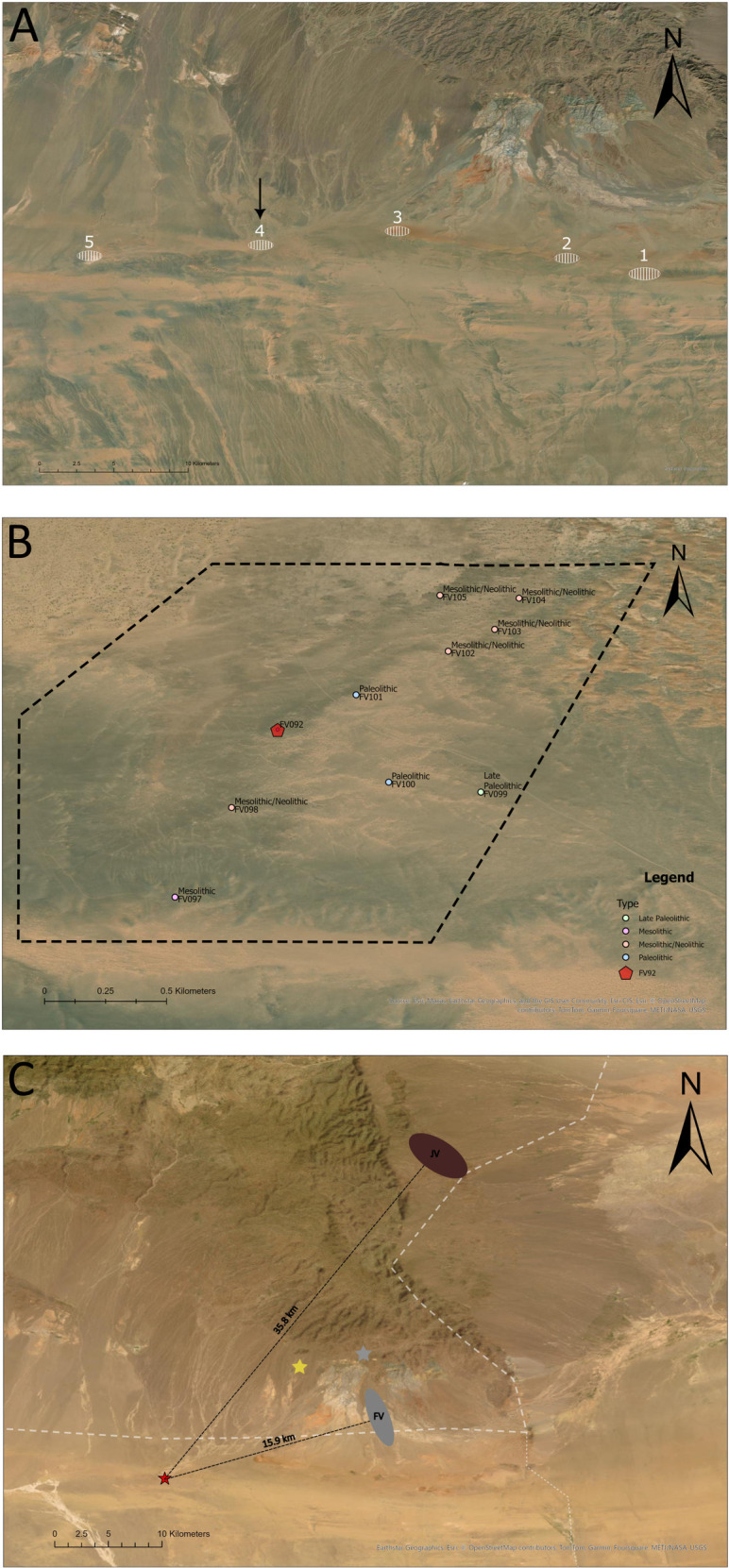
Location of paleolakes, raw materials outcrops, and site FV92 (created by authors in ArcGIS Pro and edited in GIMP 2.10.36; satellite image sources: Esri, Maxar, Earthstar Geographics, and GIS User Community, Esri, Esri CIS, © OpenStreetMap contributors, TomTom, Garmin, Foursquare, METI/NASA, USGS). A) Location of five paleolakes at the southern edge of Arts Bogd massif; B) the location of the sites discovered in the vicinity of Lake Luulityn Toirom with the chronology determined based on technological features; surveyed area marked with dashed line; C) location of the outcrops of raw material and sites: FV92 (red star), FV149 (yellow star), FV151 (grey star), JV – Jasper Valley, FV – Flint Valley.

Paleolake Luulityn Toirom is located in the central part of the Mongolian Plateau, in the eastern part of the inner-mountain depression Shereegiin Gashoon Basin (Table 1) [25,26]. It is one of the five studied paleolakes situated at the southern edge of the mountain massif Arts Bogd, constituting the easternmost fragment of the Gobi Altai Mountains (Figs 1 and 2A). Nowadays, it is the area where the dry climate of the desert-steppe zone (http://ikcest-drr.osgeo.cn/data/9be2a) [27–29], predominates with little precipitation, never exceeding 100 mm/year [30]. In geological terms, Lake Luulityn Toirom is situated south of the Main Mongolian Lineament within the Gobi-Altai Zone, constituting the lower to upper Palaeozoic accretionary wedge [31,32].

**Table 1:** Basic geographical information about Lake Luulityn Toirom.

Name of the lake	Geographical location	Height above sea level [m]	Length max. [km]	Width max. [km]	Area [km²]	Elongation
Luulityn Toirom	44°08′11” N 102°39′47” E	1260	3.40	0.93	1.83	NE-SW

### Geological studies

Geological research involved identifying the geological structure of the area surrounding the lake and the sediments that filled the paleo-reservoir. The characteristics of the sediments were determined based on material obtained from 11 hand drillings, which reached depths of 2–5 m below the surface. This enabled the determination of lithological vertical profiles of the drill holes and trench FV92, which were subsequently subjected to sedimentological analysis. The deepest probing holes (J4/1, J4/4, and J4/8) were located at the boundaries of the visible paleolake basin (Fig 1B). The changes in sedimentation conditions records were based on the material obtained in seven drill holes arranged in the NNW-SSE direction, i.e., perpendicularly to the reservoir’s axis (Fig 1B). The deepest were J4/1, J4/4, and J4/8 (Fig 1B). The first (J4/1), reaching 5 meters below the surface, was drilled in the extreme south-western part of the contemporary depression of the terrain, the second (J4/4–4.1 meters below the surface) in its north-western part, and the third (J4/8–5 meters below the surface) in the reservoir’s central part. The depth of the remaining drill holes, arranged in a southeastern direction, did not exceed 2.5 meters below the surface.

**Fig 3:**
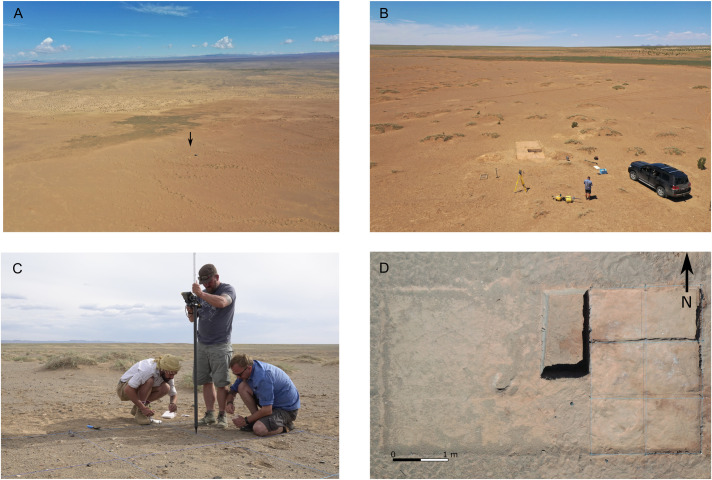
Photographs of the excavations at site FV92 during the season of 2022. A) drone photograph with the view (from southeast direction) on the Lake Luulityn Toirom and site FV92 – marked with arrow; B) drone photograph of the trench with the eastern shore of the paleolake and the Arts Bogd massif visible in the background; C) documenting the location of the artifacts discovered on the surface of the trench I/22 (from left: Grzegorz Michalec, Marcin Szmit, and Mirosław Masojć); D) photograph of the trench I/22 after completing the excavation.

Four samples were collected from drill holes J4/1 and J4/8 (Table 2) and subjected to Optically Stimulated Luminescence (OSL) dating to determine the chronology of the deposits. Their genesis was determined based on their lithology (sediment features, grain size, and color), as well as the depth and age of the deposits. Remote methods, utilizing Geographic Information System (GIS) analysis of satellite photographs, were employed to determine the extent and geometry of the lakes.

**Table 2:** OSL dating results of the samples from Lake Luulityn Toirom.

Lab. Code	Sample ID	Geographical location	Alpha content (Bq/kg)	Beta content (Bq/kg)	Gamma content (Bq/kg)	Cosmic radiation (Bq/kg)	Dose rate (Gy/ka)	Overdispersion (%)	Equivalent dose (Gy)	OSL Age (ka)
GdTL-4709	J4/1_3.0	44°07′57” N 102°39′29” E	0.02 ± 0.01	1.98 ± 0.09	0.83 ± 0.04	0.18 ± 0.02	3.00 ± 0.10	10	260 ± 10	86.6 ± 4.1
GdTL-4710	J4/1_5.0	44°07′57” N 102°39′29” E	0.02 ± 0.01	2.13 ± 0.09	0.90 ± 0.04	0.14 ± 0.02	3.19 ± 0.10	10	281 ± 10	88.1 ± 4.2
GdTL-4711	J4/8_4.80	44°08′07” N 102°39′41” E	0.02 ± 0.01	1.82 ± 0.08	0.86 ± 0.04	0.14 ± 0.02	2.84 ± 0.09	20	207 ± 18	72.9 ± 6.8
GdTL-4712	J4/11_1.0	44°08′00” N 102°39′55” E	0.10 ± 0.04	1.67 ± 0.08	1.41 ± 0.05	0.24 ± 0.03	3.42 ± 0.10	27	28.0 ± 2.7	8.13 ± 0.83

### Archaeological fieldwork

The research was conducted with the permission of the Ministry of Culture of Mongolia (permit no. A/255). Site FV92 was discovered on the surface during the prospection of the southeastern part of Lake Luulityn Toirom [21,22]. It was carried out mainly in the southern and eastern parts of the lake due to the dense vegetation and dunes in its remaining parts. The prospection was conducted by a team of five people, who surveyed the designated area at equal distances (10–25 m) over three days (Fig 2B). Newly discovered sites were assigned numbers in a growing sequence within the entire research project (https://archeo.mongolia.uwr.edu.pl/en/), while localization measurements were made using a handheld GPS device – Garmin GPSMAP 64s (Fig 2B).

FV92 is the site with a visible and dense cluster of lithic artefacts on the surface, and it is the closest to the present-day paleolake basin of all the sites discovered to date, located next to Lake Luulityn Toirom. The excavation trench at the site was opened in the place where the cluster of artifacts was identified on the surface (Fig 3). Outside the area of the set trench, stone artifacts were visible singularly or were completely absent on the surface. The excavations covered an area of 18 square meters, which was divided into sections using the local meter grid, based on the WGS 84 geodetic system. Excavations were carried out to a depth of approximately 20–25 centimeters below the surface, until sterile sediments devoid of artifacts were reached. In the central-northern part of the excavation, the trench was excavated to a depth of one meter below the surface to check the stratigraphic situation of the sediments. The artifacts discovered on the surface and during the excavation were located using a robotic total station (Topcon GT-1203) in a hybrid system with GNSS HiPer SR. The excavated sediments were dry-sieved through a 3 mm mesh. All the artifacts discovered during sieving were assigned numbers and the coordinates of square meters, mechanical level, and stratigraphy unit.

### Optically Stimulated Luminescence (OSL)

The Gliwice Luminescence Laboratory employed the optically luminescent method on four samples, as detailed by Moska et al. [33]. High-resolution Canberra gamma spectrometry, calibrated with reference materials (IAEA-RGU-1, IAEA-RGTh-1, and IAEA-RGK-1) from the International Atomic Energy Agency, was used for dose rate measurements. Dose rates were determined through an online calculator [34] incorporating the latest conversion factors. An assumed average water content of (10 ± 3%) was considered for all samples. Cosmic ray dose rates to the site were computed following Prescott and Stephan’s [35] calculations. The computed dose rate results are summarized in Table 2.

For OSL measurements, coarse quartz grains were extracted from sediment samples using routine treatment methods [33]. An automated Risø TL/OSL DA-20 reader, equipped with a calibrated 90Sr/90Y beta source delivering approximately 6.0 Gy/min to grains at the sample position, was employed for all OSL measurements. A 6 mm Hoya U-340 filter was used during OSL measurements. Final equivalent dose (De) values were calculated using the Central Age Model (CAM) or Minimum Age Model (MAM) [36] based on the dose distribution shape and overdispersion parameter. The overdispersion parameter was determined using the R package ‘Luminescence’ [37].

Analysis of the dose distribution presented in S1 Fig [38] reveals that the MAM model must be applied to two samples (GdTL-4711 and GdTL-4712) due to the multimodal character of the distribution and a high overdispersion parameter (Tables 2 and S3). The use of the CAM model is entirely justified for the other two samples (GdTL-4709 and GdTL-4710).

### Lithic and spatial studies

The analysis of lithic artifacts was based on techno-typological studies, while the typological classification itself was based on a syncretic list comprising the categories proposed by, e.g., Derevianko [12,13], Janz [10], Qu et al. [39]. All the information, including the artifacts’ technological, metric, and typological features, condition, and location, was entered into the database created in the MS Access program.

The artifacts whose maximum size exceeded 10 mm were measured using electronic calipers with a measurement accuracy of 0.1 mm. The debitage was classified as chips, which did not display the technological features of bladelets and their maximum sizes did not exceed 10 mm. Maximum lengths of the debitage were measured along the products’ central axis, based on the method proposed by Andrefsky Jr. [40]. Additionally, in the case of flakes, bladelets, and complete tools, the following parameters were measured: maximum width, maximum thickness, platform’s maximum width, and length. Measurements of cores included the following metric features: maximum length, maximum width, maximum thickness, and the platform’s maximum length and width. All the cores and selected tools were photographed. All the artifacts were weighed on electronic scales with an accuracy of 0.1 grams.

The technological analysis was based on the analytical approach known as the *chaîne opératoire*, which aims at determining technological behaviors comprising individual stages of production: from the extraction of raw material to the production of tools, their use, and discardment [41–43].

Apart from the above, a scar pattern analysis of cores and bifacial tools was carried out [44], on the basis of the objects themselves as well as photographs. The use of scar pattern analysis aimed at reconstructing the process of knapping and individual production sequences when insufficient data were acquired during the refitting studies of the material [40,44,45].

Another method used in the technological analysis was refitting studies. Apart from determining the production method and reconstruction of stages of core reduction leading to the production of blanks [44,46–48], the method was employed to verify the impact of post-depositional processes on the condition of the discovered concentration of artifacts [46,49,50]. The analysis resorted to three types of refit proposed by E. Cziesla [46]: break refit, production sequence refit, modification, and resharpening refit. The refitting studies were conducted by three individuals who worked jointly for 140 hours. The refitting stage ignored small-sized artifacts, such as chips; the remaining lithic artifacts acquired during excavation and sieving were divided into two categories: kind of raw material and type of artifact. The refitted blocks and single refits were described according to the type of refit and the core reduction stage. Selected blocks were photographed.

A spatial analysis of the location of the sites within the fourth paleolake and the artifacts within site FV92 was carried out using the ArcGIS Pro program. The number of artifacts (frequency of their occurrence within grid squares) found on the surface and during sifting of the material was totaled up and presented graphically. Spatial analyses were carried out separately for both stratigraphic units where the lithic artifacts were discovered. To check the influence of postdeposition processes, as well as to determine whether there is a primary cluster of artifacts, the following analyses were applied: a scatter of artifacts concerning their type and weight, analysis of the spatial localization of the combined elements during refitting studies, and the kernel density analysis (bandwidth – 0.25).

### Use-wear studies

Functional analyses of the lithic material from the Gobi Desert, however rare, include those concerning Holocene stone axes and adzes [51], as well as grinding and pounding tools [52]. They provided data on the exploitation of raw plant materials, enabling a better understanding of the function of the discovered artifacts. The research is part of comprehensive multidisciplinary projects [7,11] aiming at reconstructing, among other things, the ways of exploiting the natural environment of the Gobi Desert by prehistoric communities.

A total of 46 artifacts were the subject of analysis, including almost all (34 of 36) of the tools acquired during the excavations and a sample of randomly selected bladelets comprising 12 specimens. The artifacts were made of three raw materials: jasper, chalcedony, and flint. The examination aimed to record possible traces of their use remaining on their edges and surfaces. Microscope Olympus SXZ9 of magnification 6.3-57x was used to observe damage and rounding, while microscope Olympus BX53M of magnification 100-200x was used to identify polish and abrasion.

To accurately interpret the traces recorded on the edges and surfaces of the studied artifacts, experiments were conducted using a jasper concretion from the Jasper Valley (JV) region. Flakes and blades detached from the jasper concretions were used for various activities. Three flakes, including two with retouched edges and one with a raw edge, were used to scrape the dry boar skin, which was lightly colored with ochre. When one tool was no longer effective (after about an hour of work), another was used in its place. The leather was then cut into strips using four flakes. Another three flakes were used to scrape fresh softwood (birch), and three more were used to scrape fresh hardwood (oak). In both cases, they were used to remove the bark and then scraped to obtain the tool handles. Four blades were used to cut both fresh softwood and hardwood. Another activity conducted as part of the experiments was scraping fresh bones, which was done with three flakes, and cutting bones with three blades. Four blades were also used to separate the fresh meat from the bones. Seven flakes and blades were used to process plants. As part of this activity, plant fibers were split and scraped, and the cane was cut. All these activities were completed within one to two hours.

## Results

### Paleolake characteristics and stratigraphy of the lake sediments and site FV92

The analysis of the terrain relief, based on the area’s prospection and satellite photographs, proved that Lake Luulityn Toirom was an endorheic reservoir fed by watercourses draining the surrounding area. The interior basin developed in the loamy-sandy sediments of the upper-Cretaceous Baruungoyot formation (Fig 1B) [53]. The paleolake stretched for 3.4 km along the NE-SW axis, and its maximum extent occupied an area of approximately 1.83 km² (Table 1). It was mainly supplied from the north and northeast (Figs 1B and 2A) by the watercourses draining the Arts Bogd mountain massif. Smaller watercourses flowed into the lake from the south, from the ridge of an inconspicuous cuesta. The contemporary bottom of the paleolake is situated at an altitude of approximately 1260 m above sea level. The paleolake is limited from the west by a visible low escarpment. In the East, coppice (Nabkha) type dunes encroach on the flat surface of the dried-out reservoir. Aeolian sands occur along its northwestern edge (Fig 1B). The lake’s surface rises slightly towards the south, and site FV92 is located slightly higher than the contemporary bottom of the paleolake (0.5–1 m). Given the above, it is clear that the southernmost boundary of the paleo reservoir in the Pleistocene ran between site FV92 and drill hole J4/11 (Fig 1B).

Drilling in the paleolake revealed the presence of sediments connected with fluvial, limnic, and aeolian sedimentation. Eight facies (units) are present within the deposits, but their thickness in individual profiles varies. The sediments identified in individual drilling profiles also differ marginally.

**Fig 4:**
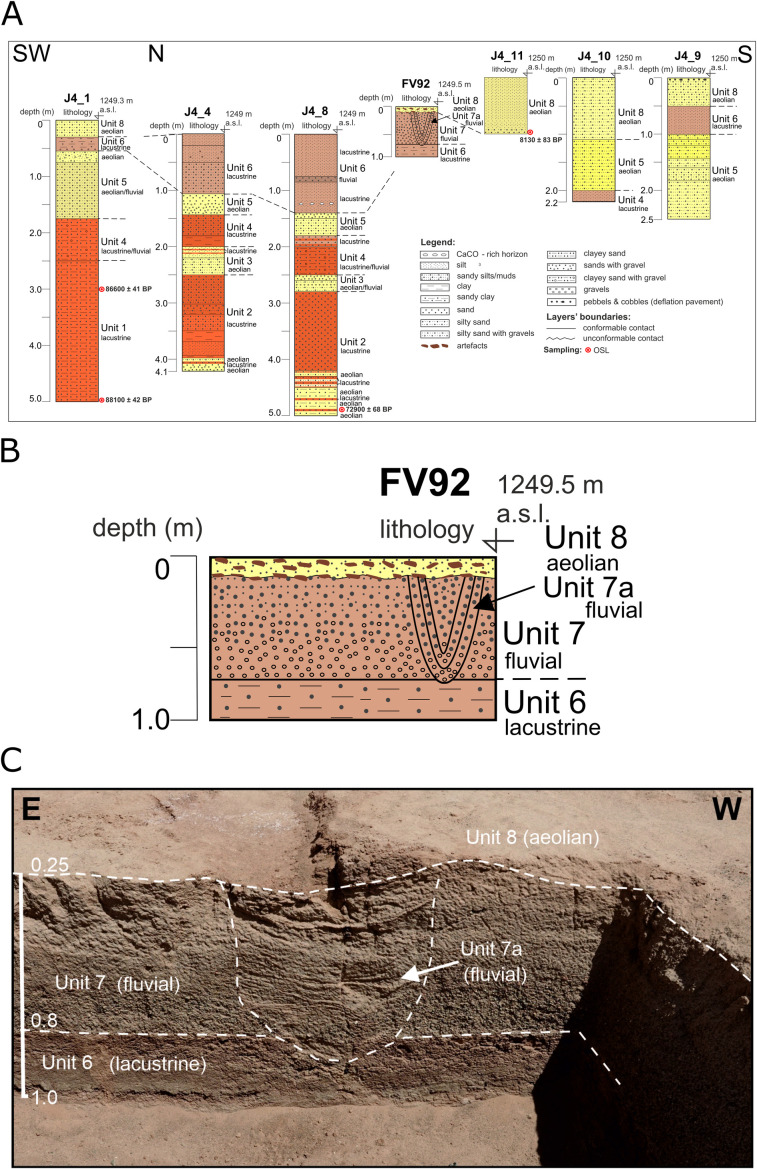
The chronostratigraphic situation of limnic deposits and sediments at site FV92. A) profiles of drill holes and archaeological trench along the transverse axis of the south-western part of Luulityn Toirom Lake; B and C) profile of the FV92 trench on the southeast bank of Luulityn Toirom Lake. View in the photo towards the NE.

The oldest Unit 1 was identified in drill hole J4/1; it consists of reddish clayey sands, with a thickness of 3.5 m (Fig 4A). The age of the deposits was determined for two samples obtained at the depth of 5 m and 3 m below the surface, which is, respectively, 88,100 ± 42 BP and 86,600 ± 41 BP (Table 2).

Younger sediments, representing Unit 2, were drilled 5 m below the surface in drill hole J4/8. The chronology of the sample of rusty-red silty sands was determined to be 72,900 ± 68 BP (Table 2), which suggests that these sediments accumulated during the cold period of MIS-4. Above them, in the interval of 4.3–4.5 m below the surface, there are yellow and rusty-red silty sands. Its counterpart in drill hole J4/4 is a thin (ca. 0.12 m) horizon of silty sands and silty sands with gravels. Above is a series of sediments in both drill holes consisting of rusty red sands, silty sands, and silts. In drill hole J4/8, the facies consists of sands at depths of 2.80–4.20 m below the surface (Fig 4A). In drill hole J4/4, it occurs at depths lower than 2.50 m below the surface, and in the interval 3.50–3.95 m below the surface, it is represented by alternating layers of silt and loam.

Unit 3 was identified in drill holes J4/4 and J4/8 (Fig 4A). In drill hole J4/4, Unit 3 reaches a thickness of up to 0.5 m (2.00–2.50 m below the surface). It consists of yellow silty sands, which in the upper part change into horizontally bedded sandy silts with two laminae of red loams. Their counterpart in J4/8, located at 2.50–2.80 m below the surface, consists of sandy silts and sands with fine gravel.

Unit 4 was identified in drill holes J4/1, J4/4, J4/8, and J4/10 (Fig 4A). It consists of rusty-red clays and silty sands (J4/4), red and brown sands (J4/8), clayey sand with gravels (J4/1 and J4/8), and brown silts (J4/10). The maximum thickness of the sequence reaches 0.7 m, which was identified in drill holes J4/1 and J4/8.

Unit 5 was identified in all the drill holes except J4/11 (Fig 4A). It consists of yellow sands and sands with gravel (J4/4, J4/8), silty sands and silty sands with gravel (J4/8, J4/9), and sandy silts (J4/8, J4/9, J4/10). The thickness of Unit 5 reaches up to 1.5 m, which was recorded in J4/9.

Unit 6 is absent only in J4/11 and 14/10. It is considerably diversified and consist of brown silty sands with gravels (J4/4), sandy clays (J4/1), sands (J4/8, FV092), gravels (J4/1, FV092), sands with gravels (FV092), silts (J4/4, J4/8, J4/9) and sandy clays (FV092; Fig 4). The thickness of this sedimentation unit reaches its maximum of 1.4 m in drill hole J4/8.

In trench FV092, Unit 7 was identified as a facies of fluvial sediments of silty sands and a channel facies of fluvial sediments (gravels and sands; Fig 4), which was identified as Unit 7a.

The youngest Unit 8 is situated at the highest level; it consists of yellow silts (S) (J4/11, F0V92), sandy silts (J4/10), silty sands (J4/1, J4/9), sands (J4/11), and pebbles (J4/9, FV092). The sequence reaches the maximum thickness of 1.10 m in J4/10 (Fig 4). The age of the deposits in this sequence was determined as 8130 ± 83 BP (Table 2) on the basis of dating the sample obtained in J4/11 (at a depth of 1.0 m).

### Lithic assemblage from FV92

The artifacts’ assemblage consists of 2726 products (Table 3). It includes ten cores and one precore. The most numerous are debitage and waste (n = 2679), among which 1711 chips, 661 flakes, 290 bladelets, and 16 chunks were identified. Moreover, 36 tools were found together.

**Table 3:** FV92, structure of lithic assemblage—number and weight (in grams) of artifact classes by raw materials.

	Jasper				Chalcedony				Flint				Opal				Quartzite				Total			
Artefact type	n	%	weight	%	n	%	weight	%	n	%	weight	%	n	%	weight	%	n	%	weight	%	n	%	weight	%
Precore	1	0.04	61.7	4.2	–	–	–	–	–	–	–	–	–	–	–	–	–	–	–	–	1	0.04	61.7	2.0
Core	8	0.29	268.8	18.4	2	0.1	116.2	11	–	–	–	–	–	–	–	–	–	–	–	–	10	0.4	385	12.7
Flake	268	9.83	599.6	41.0	299	11.0	569	51	62	2.3	153.8	76.9	2	0.07	3.8	100	–	–	–	–	631	23.1	1326.2	43.6
Bladelet	156	5.72	92.4	6.3	74	2.7	48.8	4	5	0.2	2.8	1.4	–	–	–	–	–	–	–	–	235	8.6	144	4.7
Technical products	62	2.27	121.6	8.3	19	0.7	58.2	5	4	0.1	21.6	10.8	–	–	–	–	–	–	–	–	85	3.1	201.4	6.6
Chip	855	31.36	250.7	17.1	840	30.8	264	24	16	0.6	6.1	3.0	–	–	–	–	–	–	–	–	1711	62.8	520.8	17.1
Chunks	7	0.26	30.6	2.1	6	0.2	11	1	3	0.1	15.8	7.9	–	–	–	–	–	–	–	–	16	0.6	57.4	1.9
Retouched tool	25	0.92	14.5	1.0	10	0.4	38.2	3	–	–	–	–	–	–	–	–	–	–	–	–	35	1.3	52.7	1.7
Bifacial tool	1	0.04	24.3	1.7	–	–	–	–	–	–	–	–	–	–	–	–	–	–	–	–	1	0.04	24.3	0.8
Hammerstone	–	–	–	–	–	–	–	–	–	–	–	–	–	–	–	–	1	0.04	266.8	100	1	0.04	266.8	8.8
**Total**	1383	50.73	1464.2	100	1250	45.85	1105.4	100	90	3.3	200.1	100	2	0.07	3.8	100	1	0.04	266.8	100	2726	100	3040.3	100.0

#### Raw materials.

The analyzed assemblage of lithic artifacts shows a predominant tendency to use two types of lithic raw material: the most frequently used material is jasper (50.73%), followed by chalcedony (45.85%) (Table 3). Less numerous are the artifacts made of flint (3.3%), and there were only individual objects made of opal (n = 2) and quartzite sandstone (n = 1). It is noteworthy that there are no outcrops of raw materials located in the vicinity of the site.

#### Cores.

Cores discovered within the site were most frequently abandoned at the late stage of reduction (n = 8), and one was in a residual form, which small size probably prevented the continuation of blank production.

**Fig 5:**
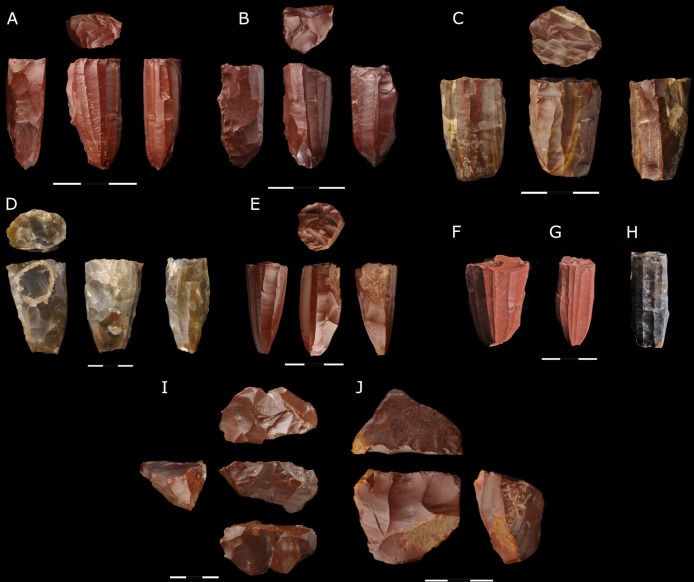
Cores. A-H) bladelet microlithic cores; I) fragment of a boat-shaped core; J) form of flake unidirectional core; cores made of jasper – A-C, E-G, I-J; cores made of chalcedony – D and H; scale 0-3 cm.

The most frequent type of core present in the assemblage is a rounded bladelet core (n = 8); three had a conical shape (Fig 5A, 5B, 5D), three were bullet (Fig 5E, 5G, 5H), and there was a cylindrical core (Fig 5C) and a pyramidal one (Fig 5F). All the forms were reduced unidirectionally. Flaking platforms display regular scars with hardly visible or, indeed, invisible traces of bulbs after flaking the bladelets. The flaking surface is usually situated all around the block and covers most of the surface of the cores. Very few forms display scars on the lateral side (n = 1) or in the back (n = 4), which appeared at the stage of preparation and forming the crest. Moreover, in three cores, slight remains of the natural surface with evidence of preparation are visible on the back. Technological features indicate that the most frequently used method of repairing cores’ striking platforms was performing a series of centripetal strikes – seven cores display centripetal scars on this surface (Fig 5A–5G). In one case, the evidence of repair employing bidirectional perpendicular strikes is visible (Fig 5H). The mean values of the cores’ maximum length, width, and thickness are 46.9 x 50.4 x 38.3 mm (Tables 4 and S2). The range of maximum length is between 38.7 and 60.1 mm. The parameters of the cores abandoned at the late stage of their reduction indicate that the blanks, whose length was between 35 and 60 mm, were produced at the final stage.

**Table 4:** Size (in millimeters) and weight (in grams) of cores.

	n	Min	Max	Mean	Median	St. dev.
Max length	8	38.7	60.1	46.9	47.5	6.46499
Max width	8	13.4	50.4	26.7	22.25	10.34878
Max thickness	8	15.3	38.3	25.7	25.2	8.047894
Weight	8	16.1	92.8	42.3	31.9	25.31477
Striking platform length	7	15.8	29.7	22.3	20.9	5.217396
Striking platform width	7	12.7	38.3	23.5	21.3	8.784053

Apart from bladelet cores, the artifact assemblage includes one multi-platform flake core made from a small jasper pebble (46.8 x 39.3 x 26.2 mm). Multidirectional scars, present on all surfaces, may result from the absence of hierarchization of production and the ad hoc method of producing small flake blanks.

#### Debitage and waste.

Debitage and waste constitute the most numerous group of artifacts, accounting for 98.3% of the entire assemblage, which includes 631 flakes, 235 bladelets, 85 technical products, 1,711 chips, and 17 chunks (Table 3). Most artifacts were made of jasper (49.4%) and a slightly smaller number of chalcedony (45.4%). Flint accounts for 3.3% of the raw material used, while individual artifacts (n = 2) were made of opal.

Flakes constitute the largest proportion of all debitage categories (n = 631). Most flakes are preserved in their complete form (n = 477), and only 26% have been fragmented (Table 5). The analysis of the contribution of the natural surface on the flakes’ dorsal face indicates that as many as 64% of the artifacts are devoid of it, and 22% have less than 50% of it. Only 12% of the products have unmodified surfaces in the range of 51–100%; for eleven artifacts, the presence of this feature was not determined. Considering the scars visible on the surface, most flakes display unidirectional scars; the next two most numerous categories, perpendicular bidirectional and multidirectional ones, constitute 18% of the flakes, each (Table 5). More than half of the flakes have plain platforms (n = 368); the next most numerous type is the natural/cortical platform (n = 61). The following types are less frequently represented: dihedral (n = 35), linear (n = 36), punctiform (n = 16), and faceted (n = 13). A considerable number had no platform or its type was not determined (n = 100).

**Table 5:** Flakes attributes by raw material.

Flakes									
Attribute		Jasper		Chalcedony		Flint		Total	
		n	%	n	%	n	%	n	%
Completeness	Complete	220	82.1	208	69.6	49	79	477	76
	Fragment	48	17.9	91	30.4	13	21	152	24
Natural surface	0%	147	54.9	203	67.9	55	89	405	64
	1-25%	42	15.7	50	16.7	4	6	96	15
	26-50%	27	10.1	20	6.7	–	–	47	7
	51-75%	9	3.4	5	1.7	2	3	16	3
	76-100%	37	13.8	16	5.4	1	2	54	9
	Unidentified	6	2.2	5	1.7	–	–	11	2
Directions of scars	Unidirectional	112	41.8	169	56.5	34	55	315	50
	Perpendicular	51	19	53	17.7	11	18	115	18
	Opposite	11	4.1	12	4.0	4	6	27	4
	Multidirectional	59	22	43	14.4	10	16	112	18
	Unidentified	33	12.3	22	7.4	3	5	58	9
Platfrom type	Natural/cortex	38	14.2	16	5.4	7	11	61	10
	Dihedral	20	7.5	9	3.0	6	10	35	6
	Linear	20	7.5	15	5.0	1	2	36	6
	Plain	151	56.3	180	60.2	37	60	368	59
	Punctiform	6	2.2	10	3.3	–	–	16	3
	Faceted	3	1.1	7	2.3	3	5	13	2
	Unidentified	30	11.2	62	20.7	8	13	100	16
**Raw material total**		**268**	100	**299**	100.0	**62**	100	**629**	100

**Fig 6:**
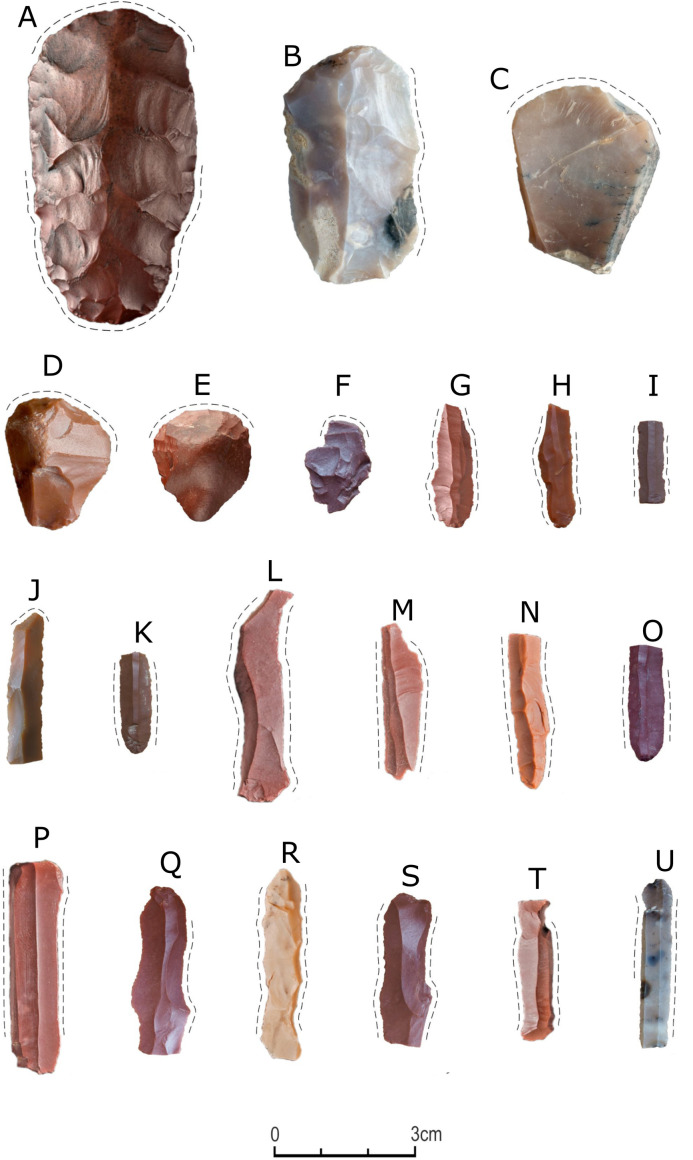
Artifacts with evidence of use (dashed line). A) bifacial tool; B-F) end-scrapers; G) perforator; H) truncated piece; I-O) retouched blades; P-U) bladelets.

Another category of artifacts comprises bladelets, the majority of which are made of jasper (n = 156), with a more negligible contribution from chalcedony (n = 74) and flint (n = 5) (Table 6 and Fig 6). It is worth noting here that the ratio of flakes to bladelets is ca. 2:1 in the case of jasper and 4:1 for chalcedony. Most bladelets were broken (n = 179), and a minority were found in complete form (n = 56) (Table 6). The natural surface is absent on the dorsal face for as many as 211 (89.8%) bladelets; only 5 display it on more than 50% of the surface. Most artifacts display unidirectional scars on the dorsal face (82.6%), and fewer artifacts display perpendicular scars (15.3%). Only three bladelets display multidirectional negatives, and two display none (cortex bladelets). As many as 91 bladelets have no platforms; their most frequently encountered types are punctiform and plain ones. Linear ones are less frequent, while one artifact has a natural platform.

**Table 6:** Bladelets attributes by raw materials.

Bladelets									
Attribute		Jasper		Chalcedony		Flint		Total	
		n	%	n	%	n	%	n	%
Completeness	Complete	35	13.1	20	6.7	3	5	58	24.7
	Fragment	121	45.1	54	18.1	2	3	177	75.3
Natural surface	0%	138	88.5	68	91.9	5	100	211	89.8
	1-25%	8	5.1	5	6.8	–	–	13	5.5
	26-50%	6	3.8	–	–	–	–	6	2.6
	51-75%	1	0.6	–	–	–	–	1	0.4
	76-100%	3	1.9	1	1.4	–	–	4	1.7
	Unidentified	–	–	–	–	–	–	–	–
Directions of scars	Unidirectional	124	79.5	66	89.2	4	80	194	82.6
	Perpendicular	28	18	7	9.5	1	20	36	15.3
	Multidirectional	3	2	–	–	–	–	3	1.3
	Unidentified	1	0.6	1	1.4	–	–	2	0.9
Platform type	Natural/cortex	–	–	1	1.4		–	1	0.4
	Linear	19	12.2	7	9.5	1	20	27	11.5
	Plain	32	20.5	16	21.6	2	40	50	21.3
	Punctiform	45	28.8	20	27.0	1	20	66	28.1
	Unidentified	60	38.5	30	40.5	1	20	91	38.7
**Raw material total**		**156**	100	**74**	100	**5**	100	235	100

Technical products – a total of 85 artifacts belonging to this group were identified. Their most significant number is made of jasper (as many as 65); those made of chalcedony (n = 19) and flint (n = 4) are less numerous. Most artifacts were crested blades from the first and second series (n = 55). The following two categories of identified artifacts are concerned with forming and rejuvenating striking platforms of cores: core tablets (n = 3) and rejuvenation flakes (n = 26). The latter have scars on the dorsal face, which are either multidirectional centripetal (n = 18) or bidirectional perpendicular (n = 8). Core tablets display unidirectional scars (n = 2) or bidirectional perpendicular ones (n = 1).

#### Tools.

**Fig 7:**
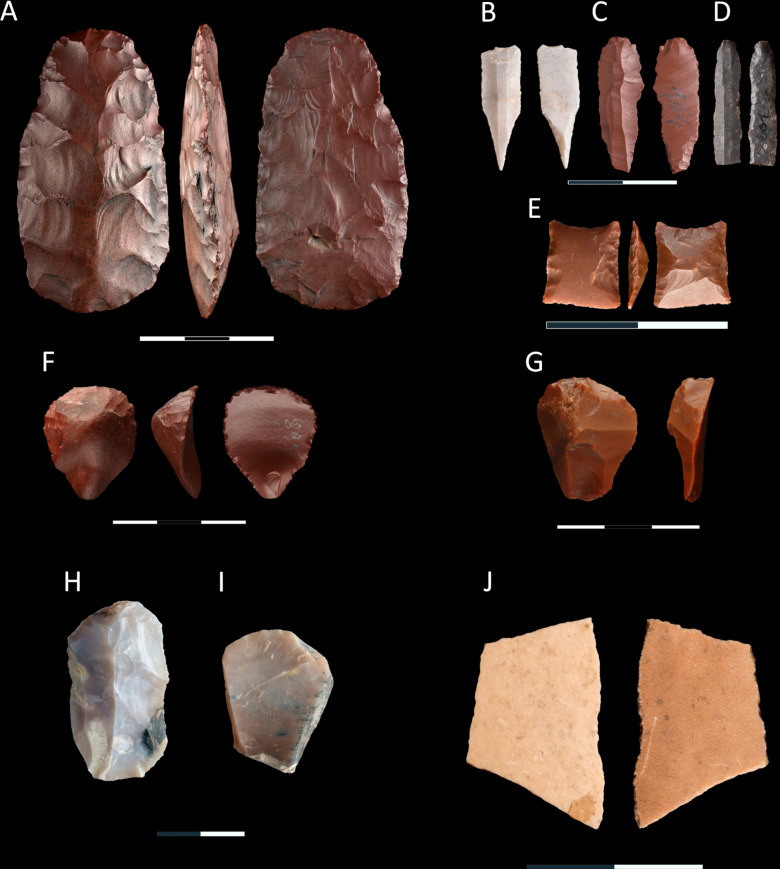
Selected lithic tools and a fragment of an ostrich eggshell were discovered at site FV92. A) bifacial adze; B and C) perforators; D) retouched bladelet; E) geometric microlith; F-I) end-scrapers; J) fragment of the ostrich eggshell; products made of jasper – A, C, D, E, F and G; artifacts from chalcedony – B, H and I; A, F-G) scale 0-3 cm; B-E, H-J) scale 0-2 cm.

A total of 36 tools were discovered in the excavated part of the site (Table 7). Most of the retouched tools were made of jasper (n = 25), while the remaining ones were made of chalcedony. As many as 60% were preserved in a complete form; the remaining are fragments of tools. Considerable differences are observed in the blanks used in the production of tools – the most popular were bladelets (n = 24), while tools made from flakes were less numerous (n = 11). Flakes were transformed into the following types of tools: endscraper (n = 6), notch (n = 2), simple retouched flake (n = 3) (Figs 6 and 7). In the case of tools made from bladelet blanks, the following types of tools were identified: retouched bladelet (n = 18), perforator/borer (n = 2), truncated bladelets (n = 2), and one geometric microlith (Figs 6 and 7). Most tools were made from the blanks that had less than 50% or no natural surface on the dorsal face, which proves that the products acquired at the late stage of core reduction were used; only one endscraper was made from a wholly cortical flake (Fig 7F).

**Table 7:** Retouched tools’ attributes by raw materials.

Retouched tools							
Attribute		Jasper		Chalcedony		Total	
		n	%	n	%	n	%
Completeness	Complete	13	37.1	7	20.0	20	57.1
	Fragment	12	34.3	3	8.6	15	42.9
Type	Retouched bladelet	16	45.7	2	5.7	18	51.4
	Retouched flake	1	2.9	2	5.7	3	8.6
	Endscraper	4	11.4	2	5.7	6	17.1
	Perforator/borer	1	2.9	1	2.9	2	5.7
	Notch	–	–	2	5.7	2	5.7
	Truncated bladelets	2	5.7	1	2.9	3	8.6
	Geometric microlithic	1	2.9	–	–	1	2.9
Natural surface	0%	21	60.0	8	22.9	29	82.9
	1-25%	–	–	2	5.7	2	5.7
	26-50%	2	5.7	–	–	2	5.7
	51-75%	1	2.9	–	–	1	2.9
	76-100%	1	2.9	–	–	1	2.9
	Unidentified	–	–	–	–	–	–
Directions of scars	Unidirectional	19	54.3	6	17	25	71.4
	Opposite	2	5.7	1	3	3	8.6
	Perpendicular	3	8.6	1	3	4	11.4
	Multidirectional	–	–	2	6	2	5.7
	Unidentified	1	2.9	–	–	1	2.9
Platform type	Natural/cortex	–	–	1	2.9	1	2.9
	Punctiform	7	20.0	1	2.9	8	22.9
	Plain	4	11.4	6	17.1	10	28.6
	Linear	3	8.6	1	2.9	4	11.4
	Unidentified	11	31.4	1	2.9	12	34.3
**Total**		**25**	71.4	**10**	28.6	**35**	100

Additionally, one endscraper was made from a rejuvenation flake of a core striking platform. Another feature of the dorsal face of the tools is the direction of scars – unidirectional negatives are the most numerous (n = 24), and less numerous are the negatives of opposing direction (n = 3), perpendicular (n = 4), and multidirectional (n = 2) ones. The last analyzed feature is the type of platform. For 12 tools, no platforms were recorded. The most numerous are plain (n = 10) and punctiform (n = 8) platforms, while linear (n = 4) and cortex (n = 1) platforms are less numerous. The mean sizes of the tools made from flake blanks are 30.1 × 23.7 × 6.1 mm, and those made from bladelet blanks are 24.9 × 7.8 × 2.2 mm. The sizes of the smallest tool, a geometric microlithic, were 9.1 × 8.1 × 2.1 mm.

The only shaped tool in the assemblage is a bifacial flaked adze made of jasper (Fig 7A). In its cross-section, it is flat-convex; the face of greater convexity displays scars of opposing direction, which originated during the shaping stage using invasive knapping. The other face, apart from numerous thinning scars of opposing and perpendicular directions, displays edge retouch on the sides and in the distal part.

#### Other lithic artifacts.

One quartzite sandstone pebble, displaying a few scars on one side, was discovered in the assemblage. The traces visible all around the surface indicate that it is a discarded hammerstone. The knapped scars were likely a result of secondary impact when a striker was used, rather than an effect of intentional reduction of the block. The artifact’s sizes are 78.4 x 68.3 x 38.3 mm.

#### Refitting studies.

The result of the refitting studies is 58 blocks, which total 144 elements. In terms of raw material, the greatest number of refits was made of jasper (n = 34), followed by chalcedony (n = 23), and one was made of flint. Two refits consisting of three and two elements, respectively, are break refits of one flake and one blade. In the remaining cases, all the refits are connected with the production sequence.

**Fig 8:**
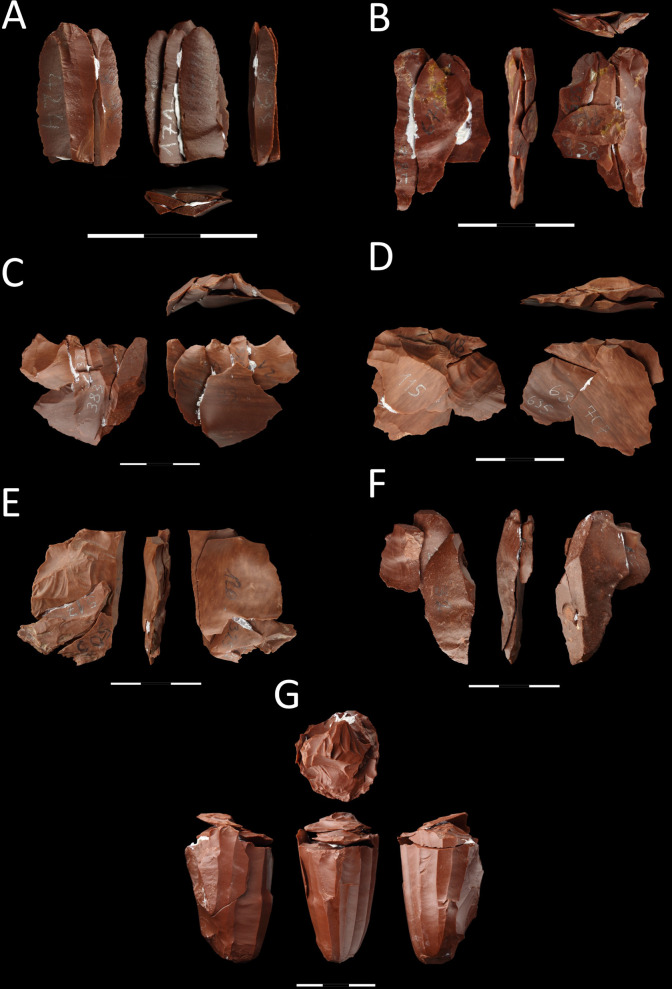
Refits with the greatest number of conjoined elements, all made of jasper. A) Refit no. 42; B) no. 13; C) no. 33; D) no. 22; E) no. 24; F) no. 32; G) no. 34.

**Fig 9:**
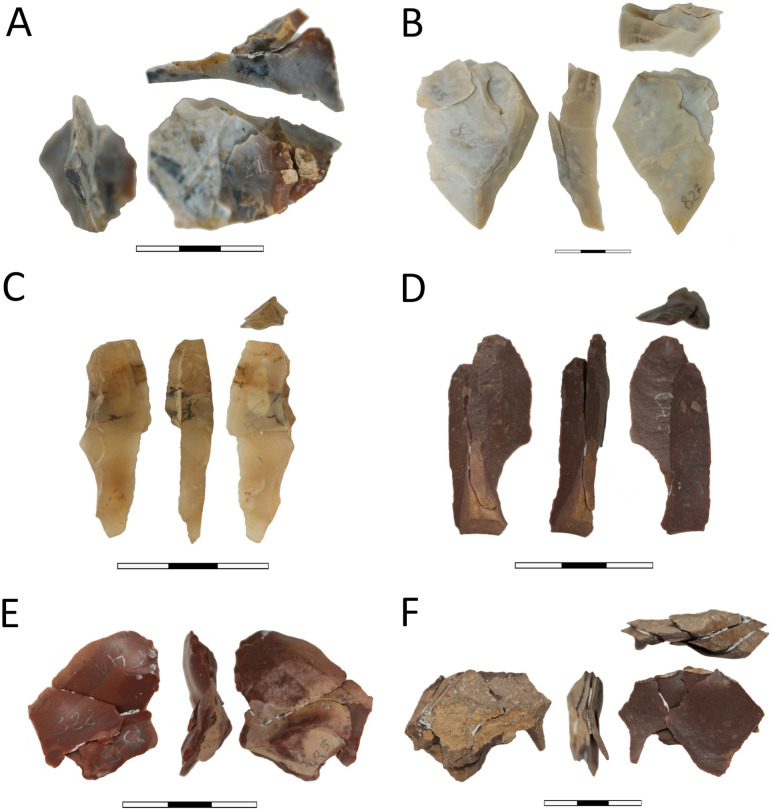
Refits comprising different stages of core reduction. A-C) made of chalcedony; D-F) made of jasper; A) refit no. 2; B) no. 11; C) no. 38; D) no. 39; E) no. 26; F) no. 20.

The most numerous group of refits consists of those representing the stage of decortication and core preparation, numbering a total of 44 specimens. The group’s most significant number of refits consists of a single refit with two elements (n = 33). Additionally, six blocks consist of three conjoined elements; one consists of four; two have five elements, and one has six conjoined artifacts (no. 33) (Fig 8C). Among the refits, including more than three elements, most represent the preparation carried out with bidirectional perpendicular knapping (n = 6), including one sequence comprising the early stage of reduction of the block’s natural surface (no. 26) (Fig 8E). Multidirectional preparation of the pre-core is visible in refits no. 22 and 33, where the structure and color of the raw material are very similar, which does not preclude the fact that the refits could have originated as elements of one block (Fig 8C and 8D). A unidirectional series of flakes which surface is mostly or entirely natural was reconstructed for block 20 (Fig 9F). The last block (no. 11) suggested the secondary removal of the crest with a series of flakes knapped perpendicularly to the main axis of the reinforced edge of the core (Fig 9B).

The stage of rejuvenating the striking platform of the core was recorded for refit number 2, made of chalcedony (Fig 9A). It consists of two flakes, one small rejuvenation flake and one core tablet knapped at a later stage. The distal part of the core tablet, which was overpassed, displays the remains of the flaking platform in the form of scars testifying to a previous stage of bladelet production and an installed crest.

The main stage of reduction of bladelet cores was recreated for ten refits, of which eight consist of two elements each, and the remaining ones include three and four artifacts, respectively. Refit number 38 represents the conjoining of a crested blade and a small bladelet made of chalcedony (Fig 9C). Another way of initiating primary production, without installing the crest and with a direct reduction of the natural surface on the flaking platform, can be seen in refits 32 and 39. They comprise individual bladelets that display a more significant portion of the natural surface on the distal face, without evidence of preliminary preparation. The remaining refits include the conjoining of completely preserved bladelets, e.g., in the biggest block, number 42 (consisting of four elements), refit of proximal parts of bladelet blanks (Fig 8B).

The last analyzed block (no. 34) consists of five elements, including one of the bladelet cores, and it represents two stages of core reduction (Fig 8G). The bladelet with a hinge in the distal part was knapped during the main production stage, followed by the repair of the core striking platform, which was carried out with a series of centripetal blows, including those executed with a larger flake, removing almost 50%. After that stage the main reduction continued, but the attempts to connect any bladelets produced at that production stage with the core were unsuccessful.

#### Experimental and use-wear studies.

**Fig 10:**
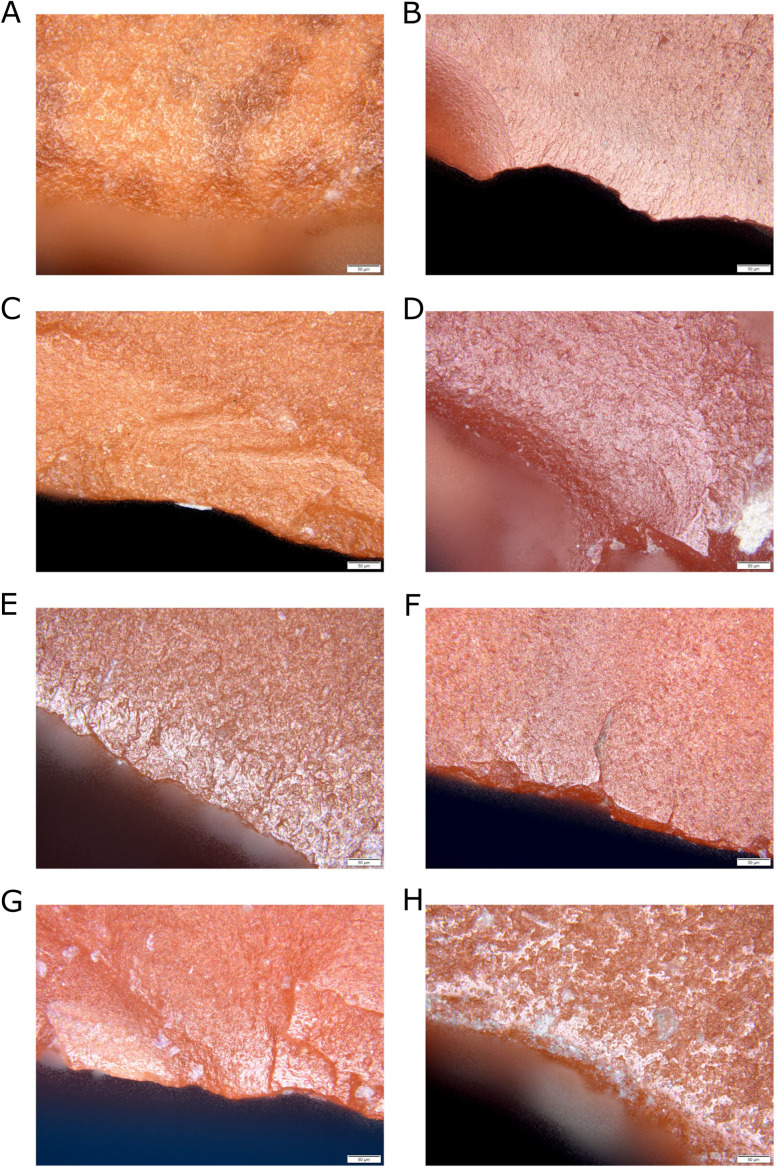
Microscopic photographs of polish traces of use observed on experimental tools, magnification 200x. A) scraping dry hides; B) scraping fresh wood; C) cutting dry hides; D) cutting fresh wood; E) scraping fresh bones; F and H) scraping fresh plants; G) cutting fresh plants.

Blanks produced for the experiment—blades and flakes—were used for various activities. Scraping hide resulted in the rounding of the working edge of the tools and the development of a dull polish (Fig 10A). Additionally, small, rounded pits and micro-craters were recorded. In the case of hide cutting, similar traces were observed; however, they appeared in bands arranged diagonally to the working edge (Fig 10C). This activity also caused small edge damages visible on the tools. Woodworking resulted in edge damage on the working parts of the tools and the development of a bright, smooth polish (Fig 10B and 10D). The polish developed more quickly when working with soft wood than with hard wood, and fewer edge damages were observed. In the case of scraping and especially cutting bone, edge damage was observed along the working edges, the intensity of which also depended on the condition of the material being worked. Moreover, very bright, patchy polish was recorded, which did not cover the entire tool surface (Fig 10E and 10G). The most intense polish was visible on the protruding edges. Residues of meat and fat on the bone caused the appearance of a brighter, “greasy” polish. Processing herbaceous plants resulted in the formation of very bright polish along the edges, depending on the activity performed—either limited to the very edge or extending further across the tool surface (Fig 10F and H). Very intense, glossy, and smooth polish traces were produced during reed processing. Additionally, linear traces in the form of fine striations were observed.

The resulting traces observed on the edges and surfaces of jasper blades and flakes exhibited the same features as those recorded on prehistoric lithic tools, which facilitated their functional identification.

Altogether, 46 artifacts were subjected to use-wear analysis, including nearly all tools discovered during the excavations (n = 34) and a sample of blades numbering 12 specimens (S1 Table). Twenty-six artifacts were made of jasper, 19 of chalcedony, and one of flint. Transformations testifying to their use were recorded for 22 artifacts (Fig 5).

**Fig 11:**
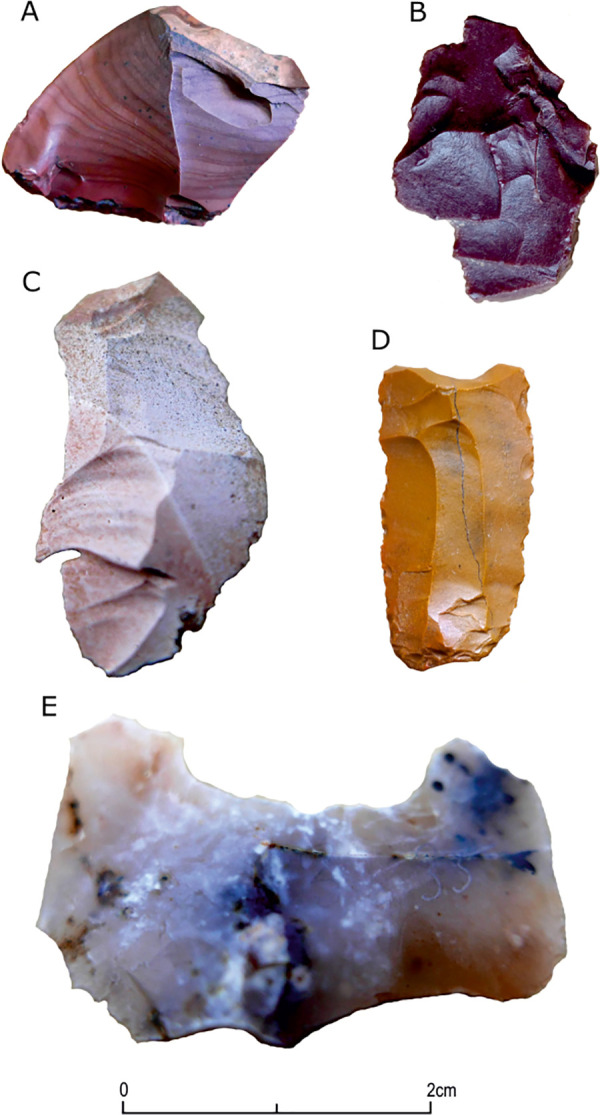
Examples of artifacts and experimental tools with natural surfaces and post-depositional traces. A) experimental tool made of jasper; B-D) displaying natural abrasion of surface, jasper; C and E) patinated artifacts from chalcedony and flint.

The surfaces of the examined artifacts displayed postdepositional (PDP) traces visible to varying degrees, resulting from being deposited in sediments or transported in deposits. Primarily, it is polishing visible with the naked eye (Fig 11B and 11D) or under magnification. In the case of one specimen made of chalcedony and one made of flint, the presence of patination was confirmed (Fig 11C and 11E). The modifications mentioned above limited the potential for observation or hindered the interpretation of the recorded traces of use, which directly influenced the lack of possibility of observing traces on six lithic artifacts. In other cases, the PDP traces did not affect the analyses.

**Fig 12:**
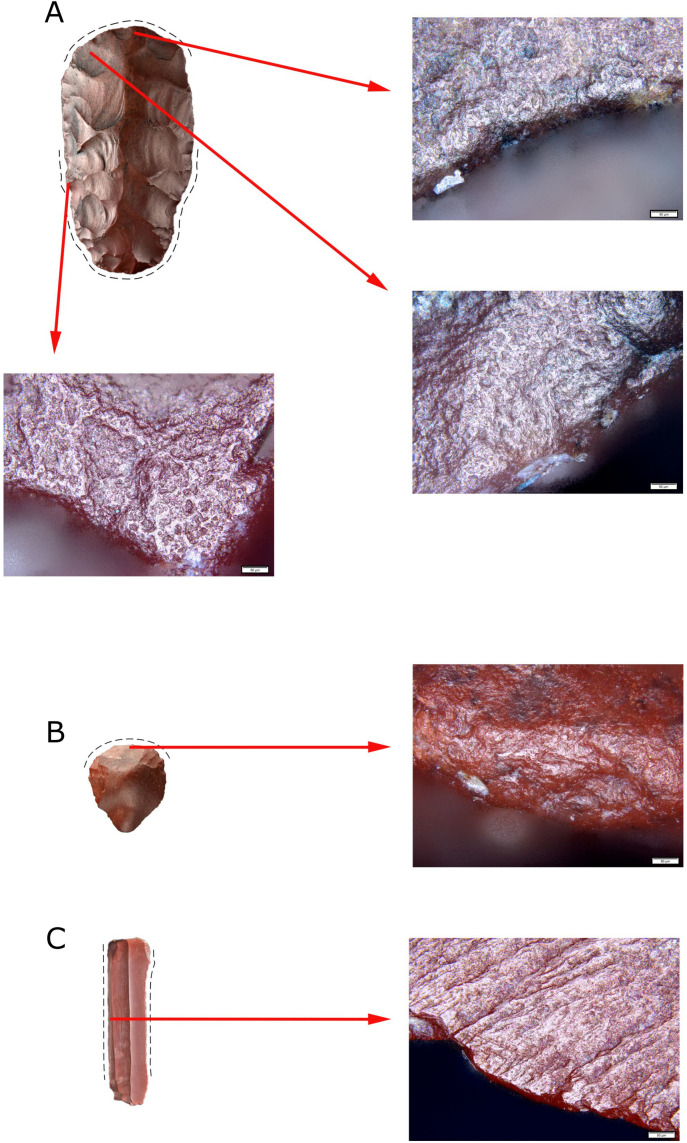
Microscopic photographs of polish traces of use. A) bifacial tool of adze type, traces of wood processing and hafting; B) endscraper, evidence of scraping hides; C) blade, traces of plant processing.

The bifacial tool was used intensively (Fig 12A). The traces of polishing is best visible on the transverse edge, resulting from wood processing. The evidence of contact with wood was also registered on the lateral edges, but it probably results from the fact that the tool was set in a wooden handle (Fig 12A).

All the endscrapers (five) subjected to analysis proved to have been used; quite importantly, one tool (Fig 6B) displayed traces of cutting, probably wood/plants, on the lateral edge. In the case of the remaining endscrapers, the changes resulting from scraping bones (Fig 6D), hides (Figs 6C and 6E; 12B), and unidentified raw material (Fig 6F) were present on the endscraper retouched edges. The nature of this evidence does not indicate intensive use of the tools. The endscrapers implemented to process hides were used at the initial stages of the work.

The traces seen on the lateral edges of the perforator (Fig 6G) prevent the unequivocal determination of the raw material processed with it. The location of the polishing traces suggests that its tip was used to drill holes, while the opposite end was set in a handle.

The function performed by the truncation remains unclear (Fig 6H); transformations were observed on the tip and both lateral edges.

Eight retouched blades displayed evidence of use. Except for one specimen, transformations were seen on the lateral edges of all the remaining ones. The tools were used for unidentified purposes (Fig 6I), except for one blade used to carve in soft raw material (Fig 6J). In this case, the traces were recorded on the tip. The retouched blades were also used to cut (Fig 6K) and process plants (Fig 6N), as well as to perform undetermined functions connected with soft and hard raw material (Fig 6L–6M and 6O).

Six of the examined bladelets displayed evidence of use on one or two lateral edges. They were used to process wood/plants (Figs 6P and 6R; 12C) and plants (Fig 6S), as well as to process (Fig 6Q) and cut (Fig 6U) soft raw material. In one case, the attempt to determine the function was unsuccessful (Fig 6T). All the bladelets display damage to the working edges in the form of breakouts.

#### State of preservation and spatial distribution.

The state of preservation of the discovered lithic assemblage is diverse. Most artifacts were preserved in a complete form (64%), and the minority were fragments (36%). When considering the state of preservation of the material itself, 94.7% of the artifacts displayed slight abrasion, perceived with the naked eye on the edges and the arrises. Very few artifacts (1%) displayed considerably abraded surfaces; in the case of 4.3% of the assemblage, which comprises only the lithics made of chalcedony, patination is seen on the surface as the change of color from transparent to matt bright-white.

**Fig 13:**
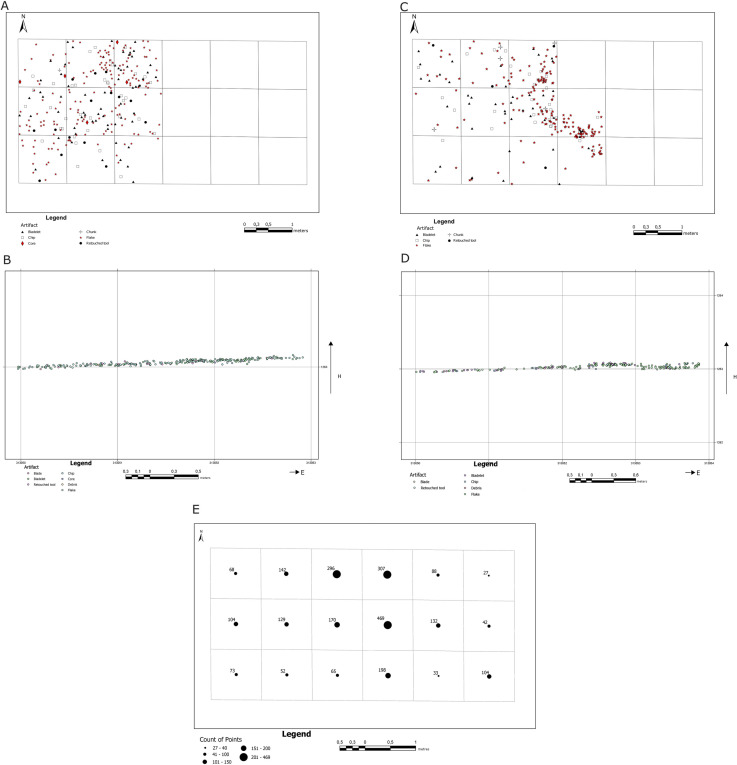
Results of analyses of the spatial distribution of artifacts. A) horizontal location of lithic artifacts with regard to artifact types within Unit 8; B) vertical location of lithic artifacts with regard to artifact types within Unit 8; C) horizontal location of lithic artifacts with regard to artifact types within Unit 7; D) vertical location of lithic artifacts with regard to artifact types within Unit 7; E) number of artifacts occurring within individual square meters.

**Fig 14:**
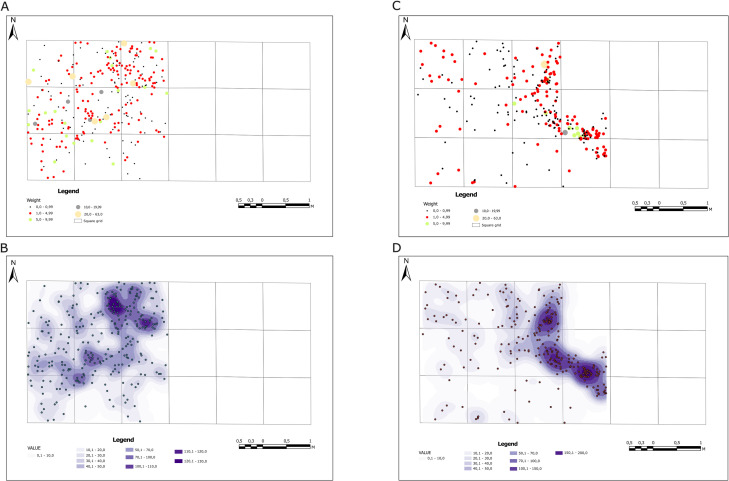
Results of analyses of the spatial distribution of artifacts at site FV92. A) horizontal location of lithic artifacts with regard to artifacts’ weight within Unit 8; B) results of the kernel density analysis for lithic artifacts discovered within Unit 8; C) horizontal location of lithic artifacts with regard to artifacts’ weight within Unit 7; D) results of the kernel density analysis for lithic artifacts discovered within Unit 7.

Artifacts were discovered within two stratigraphical units – unit 8 and the ceiling part of unit 7 (Fig 4A). The distribution of the artifacts discovered and identified within the Aeolian sediments (Unit 8) and the upper part of fluvial sediments (Unit 7) is diversified (Fig 13A–13B). The analysis of the artifacts’ horizontal location in aeolian sediments (Unit 8) reveals that bigger products with the total station were recorded only in nine square meters in the western part of the trench. It also shows that as far as their types are concerned, the location of the artifacts displays no variability (Fig 13A and 13B); the cores were discovered mainly in the northern and western parts, while the remaining categories are distributed evenly. Moreover, the analysis of the artifacts’ dispersion with regard to their weight revealed that the material was not sorted by post-depositional processes (Fig 14A). The kernel density analysis revealed a small concentration of artifacts in the trench’s northern part created secondarily by postdepositional processes (Fig 14B)

**Fig 15:**
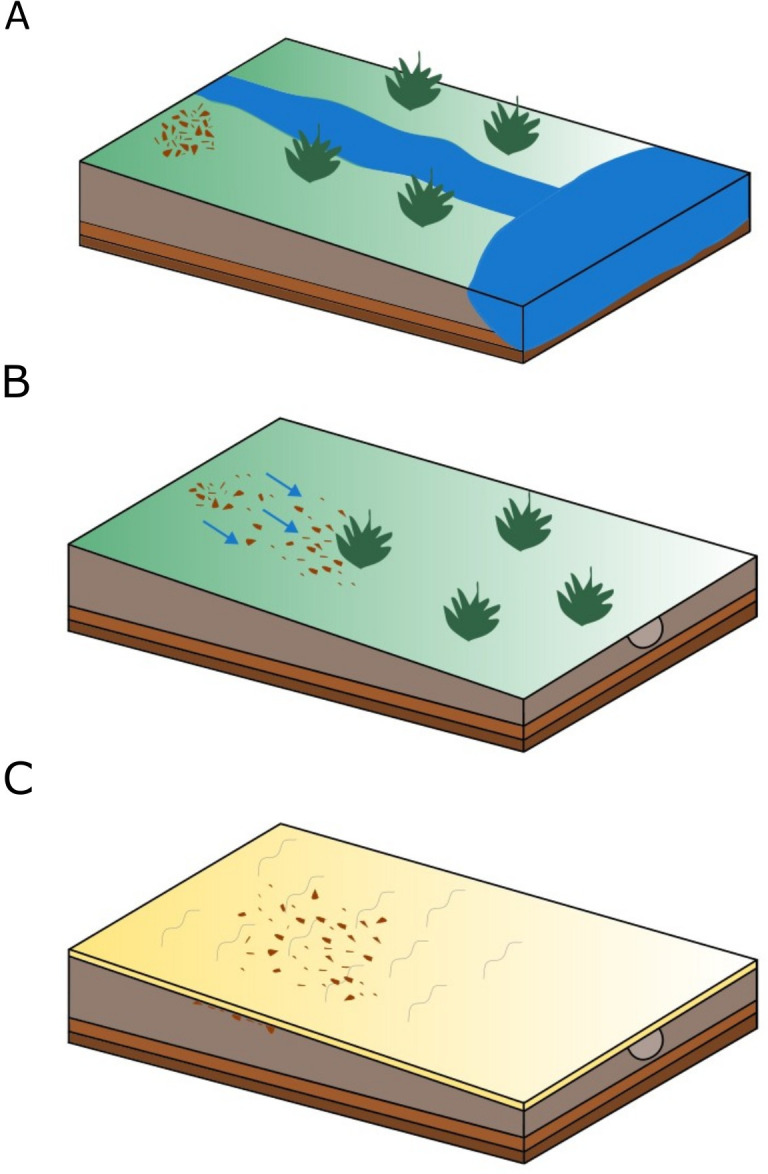
Model of the site FV92 formation process. A) location of the camp on the seasonal watercourse flowing into lake Luulityn Toirom in warm and humid conditions of the early Holocene; B) relocation of the concentration of artifacts due to seasonal precipitation and fluvial processes till the end of the boreal period; C) influence of Aeolian processes on the preservation state of site FV92 from the end of the boreal period.

The spatial distribution of artifacts in the upper part of Unit 7 differs (Figs 13C–13D and 14C–14D). Bioturbation was discovered during the excavation of the trench’s central part, and no artifacts were found within it. On the other hand, the artifacts within this stratigraphic unit were recorded in a greater area than in aeolian deposits, comprising 11 square meters. While no cores were discovered, debitage and individual retouched tools were located. The analysis of the artifacts’ weight proved that the material had been sorted – the lightest objects occurred in the trench’s western part and heavier ones in its central part, which resulted from seasonal flows in relatively humid and rainy periods (Figs 14 and 15B). The kernel density analysis revealed another difference: the present-day concentration of artifacts in the central and northern parts coincides with younger deposits (Fig 14D).

An analysis of the number of artifacts discovered during the sieving of the deposits for each square grid confirms the conclusions regarding the number of artifacts located in the excavation, with the most significant number discovered in the central and western parts of the excavation (Fig 13E).

The vertical position of the lithics confirmed the presence of a slight slope in the area – the height difference between the extreme points was approximately 15 cm (Fig 13B and 13D)

**Fig 16:**
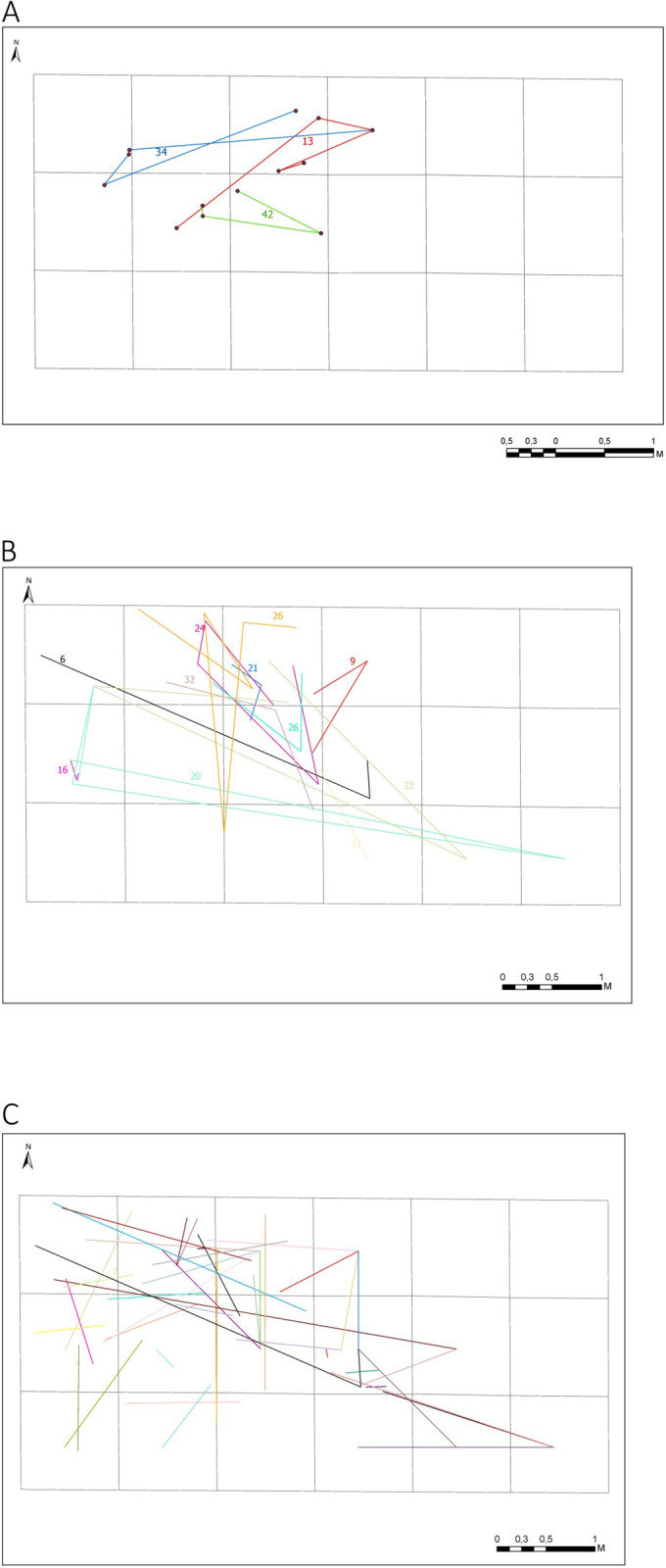
Results of spatial analyses for reconstructed blocks. A) results of spatial analyses for the blocks with the most numerous refits of elements – 13, 34, 42; B) spatial analyses for the blocks with at least three refitted elements; C) results of spatial analyses for the blocks with one refit.

The last stage in analyzing the artifacts’ spatial distribution concerned the spatial relations between the refitted elements, which totaled 58 (Fig 16). The most significant recorded distance between individual elements was over 5 meters, e.g., in the case of block no. 20, where one artifact discovered in the trench’s southeastern part fitted the ones discovered in the meters situated in the western part. The distance between the elements was usually limited to 0.5–1.5 meters (Fig 16). Rare refittings occur in close spatial relations ranging from 10 to 30 cm.

The location of refitted blocks of artifacts comprising the stage of production of bladelets and core repair (blocks no. 13, 34, 42) indicates an accumulation of elements in the north-western part of the excavation and considerable differences in distance between the refitted elements, ranging from 5 to almost 200 cm (Fig 16A.). These results should be linked to the influence of postdepositional processes on the position of the lithics and not the result of human activity itself.

In the case of refittings consisting of more than three elements, the conjoining elements were all discovered in the northern part, which reflects the previously confirmed presence of concentrations in this part of the trench (Fig 16B). Individual elements of these blocks were also discovered in the southeastern part. Considering the blocks consisting of one refit, an accumulation of refittings between the elements discovered in the northern and northwestern parts may be seen, while a smaller number of relations was observed in the trench’s southern part (Fig 16C).

### Non-lithic artifact

Apart from the assemblage of lithic artifacts analyzed above, one fragment of ostrich eggshell of sizes ca. 2 x 3 cm (Fig 7J), was discovered at the site. There is no macroscopic evidence of intentional work on the edge and surface of the specimen.

## Discussion

### Paleoenvironment interpretation and formation process of the FV92 site

The analysis of the sediments of Lake Luulityn Toirom indicates that they were formed during several accumulation phases with different time intervals. The spatial distribution of lacustrine sediments suggests changes in the reservoir’s water level, diversified sedimentation intensity types, and secondary erosion of sediments. The correlation of the sedimentological situation is more consistent for the profiles’ upper part (Units 4–8). One of the correlation factors was the color of the sediments, which is probably connected with the changes in the environment in the past. The sediments’ rusty-red color results from the iron compounds, which were facilitated by the arid climate and sedimentation in the ephemeral reservoirs of the playa type. The yellow color of the sediments may be characteristic of fluvial and aeolian sediments deposited during the cooler periods. The interpretation of profiles of the drill holes and the trench profile reveals that during the period lasting from MIS-4 to MIS-1, the lake level changed in the cooler and warmer periods and varied within the range of ca. 3 m between the reconstructed levels. Clastic material was transported to the basin of Luulityn Toirom Lake by watercourses and as a result of aeolian accumulation.

OSL dating revealed that sedimentation in the reservoir occurred as early as 88–86 thousand years ago, corresponding to MIS-5b when the climate cooled following the last interglacial (Eemian). At that time, fluvial and limnic sedimentation took place in the excavated area. In drill hole J4/1, i.e., in the reservoir’s lakefront, the series of these sediments reaches over 3 m.

The high water level present in present-day Mongolia at that time is substantiated by the results of the research carried out in the areas situated to the south of Mongolia: Qaidam Basin and Juyanze Basin [54,55], which may have resulted from the intensification of the influence of Asian monsoons (especially in the summer) on southern and western Mongolia, which was felt as early as MIS-5e [56].

The younger series of sediments identified as Unit 2 is connected with the cooling in the early Last Glacial Period (LGP – MIS-4), substantiated by the sample’s age from drill hole J4/8 (72,900 ± 68 BP; Table 2). This correlates with the glaciation present in the Khangay Mountains [57]. The sequence of sediments in Unit 2 (drill hole J4/8) revealed that thin laminates of rusty-red sands were separated by yellow-brown sands, which may testify to the material supply from various sources and of diversified origins. The roof section of Unit 2 (J4/4 and J4/8) comprises the deposition of limnic sands, clayey sands, silty sands, and silts. The common feature of the sediments in this part of Unit 2 is a distinct rusty-red color, suggesting a change of climate, warming, and drying.

Unit 3 represents an Aeolian-fluvial sequence with predominating yellow sandy silts and silts; its roof in drill hole J4/8 revealed the presence of sedimentation of sands with gravels. This indicates a local intensity of the fluvial sedimentation.

**Fig 17:**
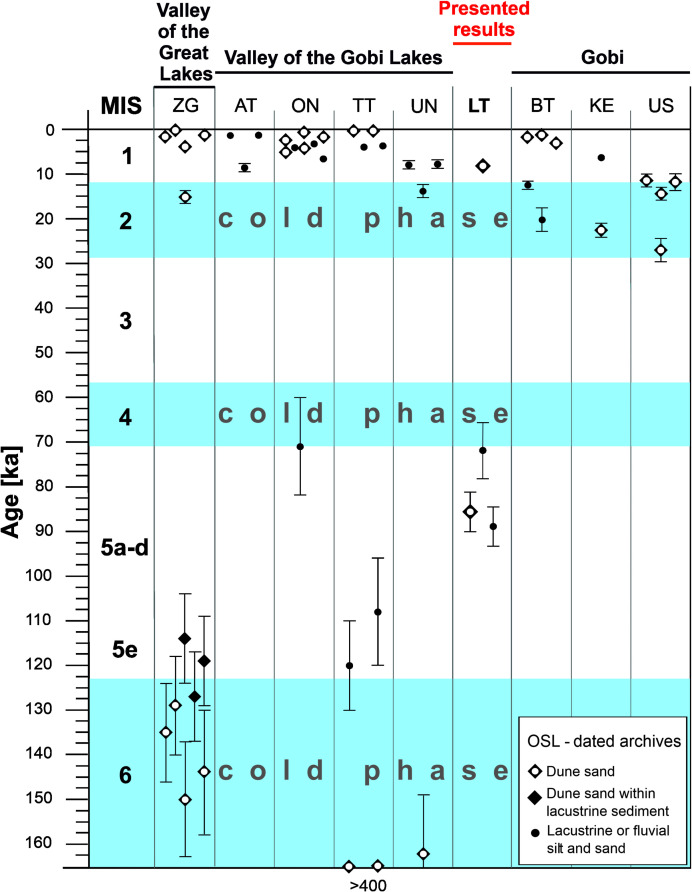
Correlation of OSL dating of lake sediments in the Gobi and Valley of Great Lakes area (changed after Lehmkuhl et al., 2018, Fig 15): Zavkhan Gol (ZG), Adagin Tsagaan Nuur (AT), Orog Nuur, (ON), Taatsiin Tsagaan Nuur (TT), Ulaan Nuur (UN), Luulityn Toirom (LT), Bayan Tohomin Nuur (BT), Khongoryn Els (KE). Ujim Sair (US).

The predominating rusty-red color of sediments in Unit 4 (silty sands, clayey sands with gravels, sands, silts, clays and sandy clays) indicates the conditions of the warm climate, more humid than that existing in the unit below and another sedimentation of the lacustrine type; this is seen especially distinctly in drill hole J4/4 at the depth of 1.4–2.1 m. The admixture of gravels in the deposits (in drill holes J4/1 and J4/8) indicates periodical fluvial sedimentation, which probably occurred during MIS-3. These data correlate with the increase of water level in Lake Orog Nuur in the Valley of Gobi Lakes, resulting from the inflow of water from the melting glaciers in Khangay (Fig 17) [58,59], as the monsoon during that period was considerably weaker than during MIS-5 [56].

Similarly to Unit 3, another facies (Unit 5) is an aeolian-fluvial sequence that appeared in the reservoirs during MIS-2 and correlates with similar phenomena in the Valley of Great Lakes and the Valley of Gobi Lakes (Fig 17) [60,61].

Sedimentation in Unit 6 (brown silts, sands, sandy clays, sandy silts, silts with gravels, and locally gravels) indicates that the climate changed again, becoming more humid, which may have taken place at the end of MIS-2 and the beginning of the Holocene MIS-1. An additional proof of increased humidity is the increase of CaCO3 in the sequence in Unit 6 (in J4/8 at the depth of 1.25 m below ground level), which may have come from the melting shells of the malacofauna. On the other hand, its source was calcium carbonate precipitating due to temporary sedimentation in the drying out reservoir of the playa type.

The profile of trench FV092 revealed local variability in the development of sedimentation in the reservoir’s lakefront from MIS-3 to MIS-1 (Fig 4). It includes sediment series connected with lacustrine, fluvial, and aeolian accumulation. The bottom of the excavated profile consists of brown lacustrine sands (Unit 6), covered by a series of fluvial sediments (Unit 7). Its profile displays a distinct gradation of grains from gravels to sands of horizontal lamination (Fig 4). This series displays evidence of erosion cuts and younger fluvial sedimentation of the gravel-sand series (Unit 7a). The profile excavated in the trench displays layered sandy-gravely deposits of the alluvial fan of the watercourse flowing from the east, probably as early as in the humid climate of the Early Holocene [62,63].

Unit 8 represents the facies developing in the lower parts of the slopes around the lake, at its southern shore. Aeolian sands of Unit 8 (J4/11) were deposited at the end of the Boreal period. They represent younger periods of the Holocene, predominated by deflationary-accumulative aeolian processes when the limnic accumulation was still developing in the reservoir. The presence of desert pavement on the surface suggests the desertification of the area and drying out of the lakes in Mongolia lasting from the late Holocene till today [64,65], which is exceptionally well seen in the Valley of Gobi Lakes to the north of the Gobi Altai [3,66]. Climate warming and precipitation reduction during the late Holocene in southern Mongolia and northern China have been confirmed by Stauch [67] and Chen et al. [68].

The state of preservation of the lithic material and the process of forming site FV92 was undoubtedly influenced by non-cultural factors such as aeolian and fluvial processes. The place of stay of the group was situated in a convenient place – close to the lake’s eastern shore and a seasonal watercourse flowing into it (Fig 7A). It is worth emphasizing here that the roof part of Unit 7 should be interpreted as a part of the living floor in the sense proposed by Dibble et al. [69], when groups of hunter-gatherers occupied this area.

On the other hand, the discovered concentration of artifacts does not reflect the encampment’s functional space [47,70,71]. It was discovered in the secondary context, while its original position was probably situated a few meters higher to the east, considering the distances between the conjoined elements of the refits. The concentration’s deposition occurred in the Early Holocene, when the climatic conditions were favorable for human settlement (Fig 15A) [1–3,72]. Then, during the humid periods of considerable rainfall, seasonal inflowing watercourses appeared, which were instrumental in transporting the artifacts along a slight slope (Fig 15B). The research results in the eastern part of the Gobi Desert proved the existence of a humid and warm Climate Optimum in the middle Holocene, resulting from the impact of the East-Asian Monsoonal system [73]. The concentration visible in the northern and central parts of the trench is the result of the influence of fluvial processes (Fig 15B); it is quite possible that the trace of bioturbation connected with the root system of a plant was another factor contributing to stopping and accumulating the flowing material in this place (Fig 13). Along with climatic changes following the boreal period (GdTL 4712–8.13 ± 0.83 Kya) [1,3,68], aeolian processes were the main factor affecting the vertical transport of the material and the surficial abrasion of the artifacts (Fig 15C). Spatial relationships between the elements conjoined during the refitting studies suggest that the discovered assemblage constitutes the evidence of a single stay. Due to the redeposition of the material, it is hard to determine whether one bigger, compact concentration or two smaller concentrations, secondarily overlapping each other.

### Technological behaviors and mobility

Due to the effect of postdepositional processes, the data obtained at site FV92 are not sufficient to determine precise subsistence strategies and mobility patterns implemented by these communities in accordance with, for example, the models proposed by Binford [74]. Yet, despite the limited data, certain preliminary conclusions may be drawn.

An attempt at reconstructing the degree of mobility may be made based on the outcrops of the raw material and its transport in the examined area [74–77]. In the close vicinity of the fourth paleolake, no outcrops of the raw materials were discovered in the area. The closest outcrop of chalcedony and flint is the Flint Valley (FV) [19,21,22], and in the case of jasper there are the outcrops situated in the area named lately – the Jasper Valley (JV), discovered as a result of archaeological prospection on the northeastern slopes of the Arts Bogd massif with more than a dozen archaeological sites of diversified provenance (Fig 2C) [22]. The JV is the only outcrop of jasper directly recorded so far. However, it is quite probable that there are more such places in the mountains. Direct evidence of raw material transport, e.g., in the form of concentrations of transported blocks of jasper, which sizes do not exceed 20 cm, were discovered at site FV 149 and FV151, situated in the valleys mouth in the Arts Bogd massif at the distance of ca. 10–15 km to the northeast of site FV92 (Fig 2C). The distances in a straight line between outcrops and site FV92 are 15.9 km for FV and 35.8 km for JV. Assuming that the mean distance covered daily by mobile hunter-gatherer communities is 20–30 km [75,78] and considering the area’s geographical setting, including the changeability of the terrain’s morphology, the access to both outcrops is diversified. The Flint Valley is not situated far, while the area separating both places is homogenous with small differences in terrain features and altitude, which facilitates access, and covering the distance should not take more than half a day. In the case of JV, apart from the distance constituting a greater logistic challenge, the area separating both locations is more diversified – the Arts Bogd massif, which is best crossed moving along the valleys situated at a maximum of 350 m higher than site FV92. Apart from geographical conditions, the mobility itself is affected by other factors, such as the size of the group and the favorable environmental conditions – a greater numerical strength of the group, as well as the humid and cooler climate, enable its members to cover greater distances and change their survival strategies [79]. An indirect indication of longer expeditions for better quality raw materials may be the discovery of traces of early Holocene settlement in the Khutul Usny Cave (FV8) [22]. This site is located by the main valley, which is the best route between site FV92 and Jasper Valley. Further research is certainly needed to confirm the hypothesis presented here, e.g., X-ray Fluorescence analysis, which would confirm the exploitation of specific raw material outcrops by hunter-gatherer communities. However, considering the distance and the logistical difficulties involved in crossing the mountainous area, it cannot be ruled out that jasper outcrops located closer to the southern slopes of the Arts Bogd Massif, which have not been located during prospecting so far, could have been exploited.

Adopting the premises proposed by Binford [74,75], site FV92 is an example of a place of special activity determined as a station, for the collector model connected with decreased daily mobility and adopting the strategy of delegating particular activities to smaller sub-groups – hunting, gathering, or acquisition of raw materials. Site FV92 should be interpreted as a small camp located at a water source that offers easy access to food and plant resources. The uncovered cluster of artifacts represents the remains of a lithic workshop, which has been preserved in a secondary position. Analyzed traces on the surface of the lithic artifacts indicate that various activities related to butchery, as well as the processing of plant raw materials, were also carried out at the site. The displacement of archaeological material from its original position does not make it possible to determine the exact zones of the activities carried out. No remains of hearts of pits were discovered within the opened trench. It cannot be ruled out that such features are located in another part of the site that was not covered by the excavation. The work results confirm this assumption carried out at the Baruun Khuree paleolake, located several kilometers to the west, where numerous Early Holocene sites bearing remains of hearths and pits have been discovered [24].

**Fig 18:**
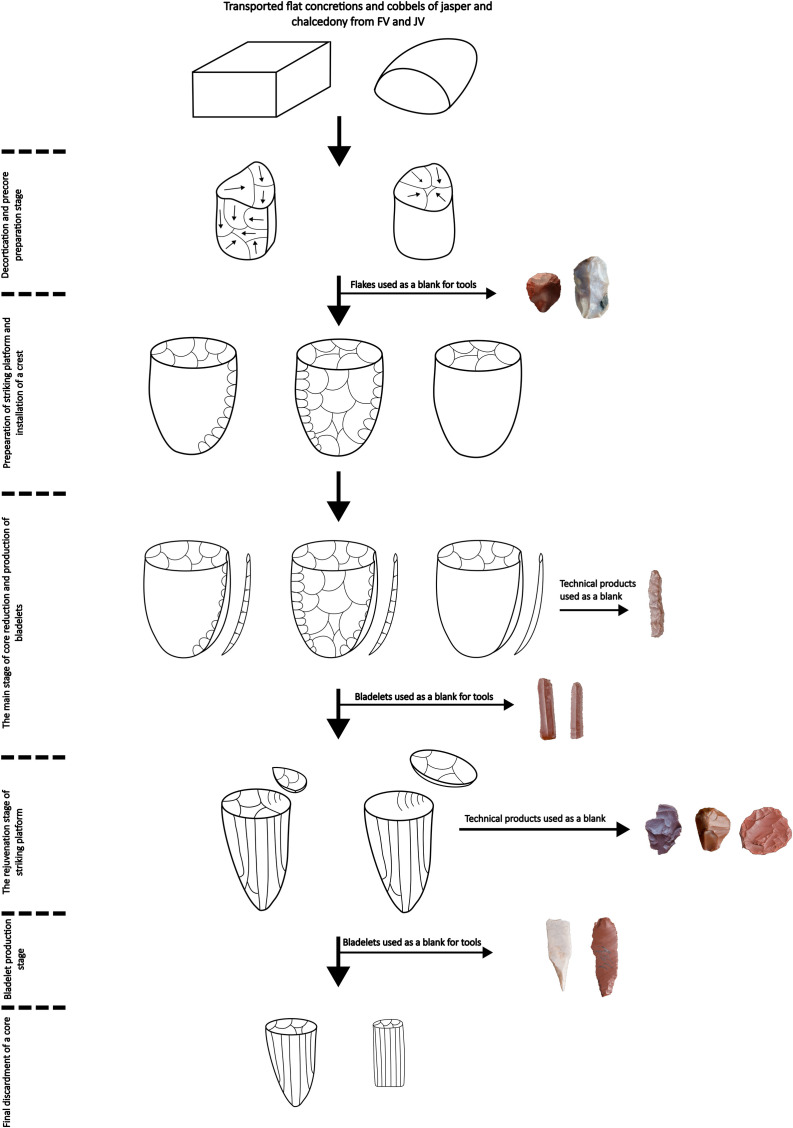
Reconstruction of individual stages of the operational chain (chaîne opératoire) of reduction of bladelet cores.

The technological picture of the assemblage of lithic artifacts discovered at site FV92 seems homogenous, while the methods used in core reduction focused on bladelet production (Fig 18). There are no premises indicating the use of opportunistic methods of flake production, e.g., with the use of unidirectional cores – the only form that could be directly connected with this activity is the form known as “precore” with individual unidirectional scars. However, the reconstructed operational chain for the method of reduction of microblade cores revealed specific differences and similarities present at individual stages of production. First, the raw material was brought in the form of concretions or cobbles, which had not been subjected to preliminary processing at all or had been only superficially prepared, as substantiated by a significant proportion of flakes displaying a natural upper surface and the conjoined refits from the early phase of reduction. The raw material used to produce rounded cores was small-sized flat or lenticular blocks/cobbles. There is no evidence of using flakes as blanks for cores. The first variant consisted in preparing the core’s platform with a series of centripetal or perpendicular strikes and preparing the flaking platform by installing a single crest or two on the opposite sides of the block. The other way of preparing a block before the main reduction was less complicated. It exploited the surface’s natural shape (convexity), similarly to the previous variant, the platform was prepared with a series of blows. At the same time, there was less interference with the surface, constituting a future flaking platform, that remained natural (Fig 18). The flakes acquired at this stage of production were exploited as raw material for producing domestic tools: end-scrapers, scrapers, retouched tools, etc. At this stage of main decortication and core preparation, the implemented technique employed direct percussion with a hard or soft hammer.

The next stage consisted of the main reduction of cores, focusing on the production of bladelets, whose edges were modified, and simple domestic tools or implements of hunting weapon – geometric microliths – were created. Cores have parallel and regular scars on flaking surfaces created during the detachment of bladelets. The extracted bladelets have mainly punctiform and linear platforms, without a strongly marked bulb and clearly visible accidental removals on the ventral face in the proximal part. The characteristics of the cores and blanks indicate the use of the pressure technique at this stage [80]. The small size of the cores, the frequent striking platform rejuvenation, and the isolated and small size platforms indicate that the specific reduction of the cores may have been carried out according to Pelegrin [80] – potentially mode 2–4, using handheld baguette or short/long crutch pressure technique was used at this stage. The main reduction of cores began with flaking a crested blade, which was continued all around or with flaking a few bladelets with natural surface. A substantial contribution of crested blades in the assemblage and a small proportion of bladelets with natural surface indicate that complex preliminary preparation of the block, which included reinforcing and shaping the edge in the form of a crest, was more readily resorted to. On the other hand, individual complex sequences representing the stage of initiating reduction without the installation of the crest indicate that this pattern was less frequently implemented at the beginning of the stage of the main reduction of bladelet cores.

Two operational patterns were also identified for repairing striking platforms. They were repaired with a series of slight centripetal or perpendicular strikes. If this repair strategy failed, e.g., due to the appearance of hinge and an inappropriate core angle, a core tablet was removed with a single blow, thus removing the original surface of the striking platform, which was later formed with a series of small removals. The technical products like core tablets and rejuvenation flakes were used as blanks for the production of retouched tools.

A fragment of a boat-shaped core was discovered in the analyzed assemblage of artifacts from site FV92. It is a failed technical product flaked during the first phase of forming the striking platform. The bottom side displays a recess resulting from the presence of a natural crack in the block.

Edge modification of retouched tools was created using two techniques. Some tools, such as the endscraper with small regular and parallel scars and the geometric microlith, were created using the pressure technique. Most tools, in the form of scrapers, retouched flakes, and retouched bladelets, were modified using the direct percussion technique. There is no evidence, either in the form of discarded microburins or the creation of notches in bladelets, crested bladelets, and trapezes/triangles, for using the microburin technique. Only two truncated bladelets and a geometric microlith could be produced using this technique.

Despite the presence of a flaked adze in the assemblage, no flakes originating from the production process of this type of tool were discovered; none of the refitted sequences can be connected with any of the stages of obtaining this type of product either. It should be assumed that this form was brought to the site as a finished product made somewhere else.

The scope of information related to the functional interpretation of tools from site FV92 is limited due to the size of the sample analyzed. The analysis results indicate that several basic raw materials were processed at the site, i.e., hide, wood, non-timber plants, and bone. The proportions of the occurrence of the different types of traces do not necessarily reflect the preference for processing individual materials. However, they provide information about the varied activities carried out within the site. The use of the analyzed specimens as elements of hunting weapons was not unequivocally confirmed during the analysis. On the other hand, traces associated with butchery activity and bone processing point to hunting as the main source of food acquisition. In contrast, traces associated with using plant materials indicate that plants are used to produce everyday items rather than as a main food source.

The tools differ in terms of the intensity of their use. Endscrapers were relatively least intensively used, contrary to the bifacial tool, retouched blades, and the blades without retouch. The tools were often set in handles. The presence of traces interpreted as the traces of hafting was found on the bifacial tool and the endscrapers. One of the endscrapers was perpendicularly hafted, which is substantiated by damages and slight retouch along its lateral edge. This suggests that the tool was used in a unique way; the remaining endscrapers were hafted in a “classic” way, i.e., the proximal part opposite to the end-scraper front were set in the handles. The perforator also was hafted. The other categories of tools were used without handles, or the hafting traces were not preserved.

### Chronology of the settlement at FV92

Based on the sediment dating results and the stratigraphic analysis, the chronological range of human activity at site FV92 should be determined as early-middle Holocene. The OSL chronology of a sample of aeolian sand determined as 8,130 ± 83 BP (GdTL-4712) is interpreted as the terminus ante quem for human settlement, while the youngest fluvial deposits are connected with humid climatic conditions of the Early Holocene. The techno- typological features of the discovered assemblage match technological behaviors recorded at other sites in Mongolia dated to that period [7,10,12].

Production of microblades in Mongolia commenced as early as the early Upper Paleolithic [14,81–84]. The beginnings and development of microblade pressure technology are connected with the period lasting from the LGM (Last Glacial Maximum) to the terminal Pleistocene [85]. Contrary to other regions of Northeast Asia, microblade technology was also used in Mongolia in the middle and late Holocene [7], which is due to the long existence over time of the hunter-gatherer model of subsistence, late domestication of animals, and the transition to the pastoral model.

Site FV 92 should be connected with phase Oasis 1/Mesolithic in accordance with the division of chronological phases proposed by Janz [7,10]. This phase is dated from the late Pleistocene (13.5 cal kyr BP) to 8.0 cal kyr BP; technologically, it characteristically focuses on orientating bladelet production with the use of boat-shape reduction methods and conical cores, with the practically insignificant contribution of deliberate production aiming at acquiring flake blanks. No pottery fragments were discovered at the site, even though they were found at the sites connected with phase Oasis 1 – fiber-tempered cord-marked pottery, only superficially fired [7,10]. The element that does not fit the picture of Mesolithic assemblages is the presence of a flaked adze; the tools of this type are mainly present in assemblages Oasis 2/ Early Neolithic dated to 8.0–5.0 cal kyr BP [7].

A few sites (FV133, 134A, 139A, 139B, 139C) were discovered and explored within the fifth paleolake in the Flint Valley [24]. The sites provided concentrations of lithic artifacts, remains of hearths, small size pits containing pottery fragments as well as individual fragments of an ostrich eggshells and products made from them – beads and pendants The assemblages of lithic artifacts display technological features similar to FV92 – use of microblade pressure technology accompanied by the presence of different types of rounded cores. Also, the raw material most frequently present at the sites in this area was chalcedony and jasper. The distinguishing element present in the assemblages is the pottery with impressed ornaments in various colors: from light gray to beige-gray to reddish. The charcoal samples were dated with the C14 AMS method to 9,810 ± 50–9,330 ± 50 BP

Another significant chronological marker in assemblage FV92 is the presence of the ostrich eggshell. Dating fragments of the ostrich eggshells from site Shabarakhusu (Bayan-dzak) localities 2 and 7 with the use of the AMC C14 method suggests human activity in the Early Holocene and secondary use of late-Pleistocene fragments of ostrich eggshells discovered on the surface by hunter-gatherer communities [17]. Technologically, these assemblages correspond to the transition period from the Mesolithic to the Neolithic [17].

Technological features of the assemblage from site FV92 closely correspond to the assemblages of artifacts discovered at site Chikhen Agui [12]. The site is a rock shelter where three archaeological horizons were discovered. These provided numerous remains of hearths, dated using the C14 method, to the period from 11,545 ± 75–5,630 ± 220 BP. The recent C14 dating results of beads made of ostrich eggshells indicate seven episodes of human settlement dating from 13,675–7,680 cal BP [15]. Identically, as at FV92, the raw materials used in the production process were chalcedony and jasper. The production methods were limited to conical, wedge-shaped and narrow-faced cores for bladelets. In the assemblages, apart from the debitage in the form of flakes from core preparation and bladelets, there are technical products – rejuvenation flakes and crested blades. Regarding tools, in the case of horizons 1 and 2, retouched bladelets predominate, while domestic tools in the form of end-scrapers, scrapers, and perforators/borers are rare. Geometric microliths and projectile points, as elements of hunting weapons, represent only a small proportion of products.

## Conclusions

The major significance of the research results presented above lies in showing the investigation potential of the vast areas of the Gobi Desert, where, thanks to our systematic field studies, traces of human activity in the Early Holocene were demonstrated in the proper geological and chronological context. The presented site, FV92, is one of many studied in this area, the chronology of which extends from the Late Pleistocene to the Middle Holocene and shows this part of the Gobi Desert bordering the Altai mountains as attractive for human settlement in climatically hospitable periods. Until recently, the agglomeration of prehistoric settlements around Flint Valley has provided important sources of camp remains – hearths, lithic and organic artefacts, and portable art objects – making this region important on the map of Central Asian prehistory [22].

The excavations at site FV92 revealed the presence of concentrations of artifacts, mainly in contemporary aeolian sediments and partly in the upper part of the older layer of alluvial deposits. Analysis of the lacustrine sediments showed the presence of the Paleolake Luulityn Toirom in different time periods from the Late Pleistocene to the Early Holocene. Relevant from the perspective of human-environment relationship studies are the results of the early Holocene, which prove the presence of favorable conditions for human settlement in the Gobi area during this period. Previous studies from the Gobi area have mainly confirmed extensive human settlement during the Middle Holocene Climatic Optimum Period and the impact of the EASM on environmental conditions [6,11].

The spatial location analysis of artifacts proves that the discovered concentration results from the influence of postdepositional processes that began in the early Holocene and do not correspond to the original functional zone. The research results presented here show the great potential of studying archaeological sites dated to the Holocene in the Gobi area, which are mostly discovered in secondary position or directly on the surface due to the influence of aeolian processes in an arid environment since the Middle Holocene. The discovered remains of human activity in the form of a concentration of lithic artifacts should be interpreted as a short-lasting encampment situated close to the lake’s shore and a seasonal tributary flowing in humid climatic conditions of the Early Holocene. The location of the raw material outcrops indicates the significant mobility of the early Holocene groups. The results of techno-typological analyses and refitting studies of the lithic assemblage show microblade technology and pressure technique use in core reduction. In addition to bladelets, flakes were used as blanks to produce domestic retouched tools. Flakes were extracted during the core preparation and repair phases. The use-wear analysis of the tools and bladelets provides evidence of using lithics for butchery activities, bone processing, and the processing of plant materials. The reconstructed picture of technological behavior fits into the Mesolithic/Oasis 1 phase (13.5–8.0 cal kya BP), according to the chronological classification proposed by Janz [7,10].

## Supporting information

S1 Fig Results of dose distribution are presented for some samples (GdTL-4709, GdTL-4710, GdTL-4711, GdTL-4712). (TIF)

S1 Table Results of use-wear studies, including number and type of artifact, presence of traces, type of activity, and material. (DOCX)

S2 Table Bladelet core measurements – raw data. (XLSX)

S3 Table Results of dose distribution – raw data. (XLSX)

S1 File Inclusivity in global research – questionnaire. (DOCX)
