# Supplementary figures and images for: Geoarchaeological research on site formation process, paleoenvironment, and human behaviors in the early Holocene of the Gobi Desert, Mongolia
Source: PLoS One. 2025 Sep 2;20(9):e0330209. doi: 10.1371/journal.pone.0330209 (PMC12404557; doi:10.1371/journal.pone.0330209)

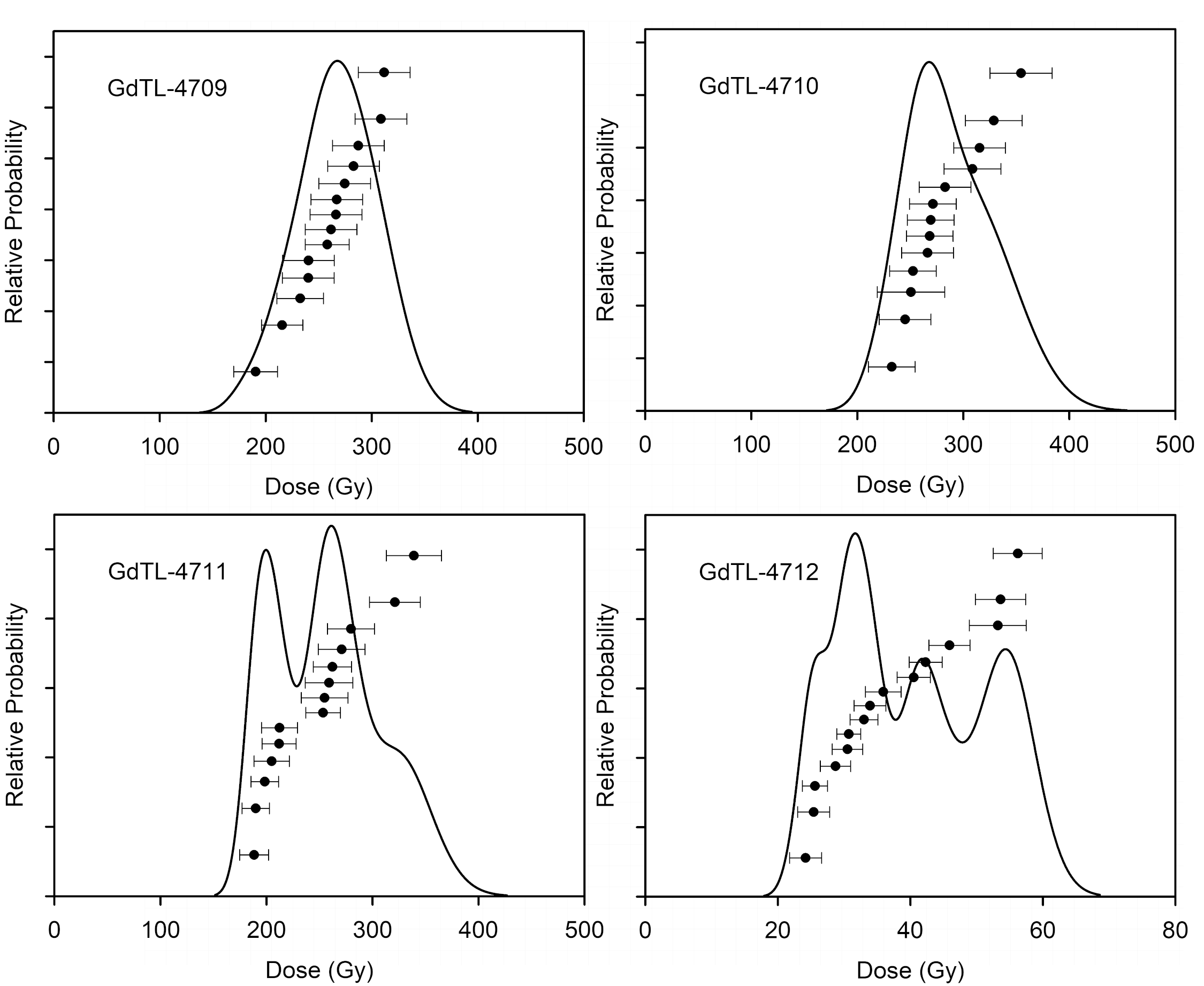

Supplement: S1 Fig — (TIF) [file pone.0330209.s001.tif]
