# Supplementary material for: Geoarchaeological research on site formation process, paleoenvironment, and human behaviors in the early Holocene of the Gobi Desert, Mongolia
Source: PLoS One. 2025 Sep 2;20(9):e0330209. doi: 10.1371/journal.pone.0330209 (PMC12404557; doi:10.1371/journal.pone.0330209)
Supplement: S1 Table — (DOCX) [file pone.0330209.s002.docx]

| **Artefact no** | **Type** | **Traces of use** | **Activity** | **Material** | **Figure** |
| --- | --- | --- | --- | --- | --- |
| 896 | Bifacial tool | Yes | Processing | Wood | 6A |
| 659 | Endscraper | Yes | Cutting | Wood/plants? | 6B |
| 278 | Endscraper | Yes | Scraping | Hide | 6C |
| 412 | Endscraper | Yes | Scraping | Bone | 6D |
| 895 | Endscraper | Yes | Scraping | Hide | 6E |
| 771 | Endscraper | Yes | Undetermined | Undetermined | 6F |
| 35 | Notch | No |  |  |  |
| 273 | Notch | No |  |  |  |
| 282 | Retouched flakes | No |  |  |  |
| 267 | Retouched flakes | No |  |  |  |
| 576 | Retouched flakes | No |  |  |  |
| 154 | Perforator/borer | No |  |  |  |
| 134 | Perforator/borer | Yes | Undetermined | Undetermined | 6G |
| 138 | Truncated bladelet | No |  |  |  |
| 264 | Truncated bladelet | Yes | Undetermined | Undetermined | 6H |
| 773 | Retouched bladelet | Yes | Undetermined | Undetermined | 6I |
| 603 | Retouched bladelet | No |  |  |  |
| 693 | Retouched bladelet | No |  |  |  |
| 737 | Retouched bladelet | No |  |  |  |
| 666 | Retouched bladelet | Yes | Scraping | Soft | 6J |
| 446 | Retouched bladelet | Yes | Cutting | Plants? | 6K |
| 330 | Retouched bladelet | No |  |  |  |
| 235 | Retouched bladelet | Yes | Working | Hard | 6L |
| 181 | Retouched bladelet | Yes | Undetermined | Soft | 6M |
| 898 | Retouched bladelet | No |  |  |  |
| 220 | Retouched bladelet | No |  |  |  |
| 913 | Retouched bladelet | No |  |  |  |
| 25 | Retouched bladelet | Yes | Processing | Plants | 6N |
| 897 | Retouched bladelet | Yes | Undetermined | Undetermined |  |
| 43 | Retouched bladelet | Yes | Undetermined | Soft | 6O |
| 165 | Retouched bladelet | No |  |  |  |
| 601 | Retouched bladelet | No |  |  |  |
| 921 | Retouched bladelet | No |  |  |  |
| 602 | Truncated bladelet | No |  |  |  |
| 92 | Bladelet | Yes | Processing | Wood/plants | 6P |
| 105 | Bladelet | No | Processing |  |  |
| 89 | Bladelet | Yes |  | Soft | 6Q |
| 20 | Bladelet | Yes | Processing | Wood/plants | 6R |
| 121 | Bladelet | No |  |  |  |
| 288 | Bladelet | Yes | Processing | Plants | 6S |
| 657 | Bladelet | No |  |  |  |
| 292 | Bladelet | Yes | Undeterminated | Undeterminated | 6T |
| 58 | Bladelet | Yes | Cutting |  | 6U |
| 174 | Bladelet | No |  | Soft |  |
| 287 | Bladelet | No |  |  |  |
| 356 | Bladelet | No |  |  |  |

**Table S1. Results of use-wear studies, including no. and type of artefact, presence of traces, type of activity and material.**
